# Multiple Myeloma Inhibitory Activity of Plant Natural Products

**DOI:** 10.3390/cancers13112678

**Published:** 2021-05-29

**Authors:** Karin Jöhrer, Serhat Sezai Ҫiҫek

**Affiliations:** 1 Tyrolean Cancer Research Institute, Innrain 66, 6020 Innsbruck, Austria; karin.joehrer@tkfi.at; 2 Department of Pharmaceutical Biology, Kiel University, Gutenbergstraße 76, 24118 Kiel, Germany

**Keywords:** STAT3, MM-CSC, antimyeloma, proteasome inhibitor, cancer, leukemia, polyphenolic, flavonoid, caspase, tumor

## Abstract

**Simple Summary:**

Multiple myeloma is the second most common hematological cancer and is still incurable. Although enhanced understanding of the disease background and the development of novel therapeutics during the last decade resulted in a significant increase of overall survival time, almost all patients relapse and finally succumb to their disease. Therefore, novel medications are urgently needed. Nature-derived compounds still account for the majority of new therapeutics and especially for the treatment of cancer often serve as lead compounds in drug development. The present review summarizes the data on plant natural products with in vitro and in vivo activity against multiple myeloma until the end of 2020, focusing on their structure–activity relationship as well as the investigated pathways and involved molecules.

**Abstract:**

A literature search on plant natural products with antimyeloma activity until the end of 2020 resulted in 92 compounds with effects on at least one human myeloma cell line. Compounds were divided in different compound classes and both their structure–activity-relationships as well as eventual correlations with the pathways described for Multiple Myeloma were discussed. Each of the major compound classes in this review (alkaloids, phenolics, terpenes) revealed interesting candidates, such as dioncophyllines, a group of naphtylisoquinoline alkaloids, which showed pronounced and selective induction of apoptosis when substituted in position 7 of the isoquinoline moiety. Interestingly, out of the phenolic compound class, two of the most noteworthy constituents belong to the relatively small subclass of xanthones, rendering this group a good starting point for possible further drug development. The class of terpenoids also provides noteworthy constituents, such as the highly oxygenated diterpenoid oridonin, which exhibited antiproliferative effects equal to those of bortezomib on RPMI8226 cells. Moreover, triterpenoids containing a lactone ring and/or quinone-like substructures, e.g., bruceantin, whitaferin A, withanolide F, celastrol, and pristimerin, displayed remarkable activity, with the latter two compounds acting as inhibitors of both NF-κB and proteasome chymotrypsin-like activity.

## 1. Introduction

Multiple myeloma (MM) is a clinically heterogeneous plasma cell cancer that is mostly restricted to the bone marrow. It accounts for almost 10% of all blood cancers and shows considerable variations in the response to treatment [1]. This leads to overall response rates that vary from a few months to more than a decade [2]. As the disease progresses, patients suffer from a repeating pattern of remission and relapse as they cycle through different therapeutic options. Typically, remission periods become shorter and tumor cells increase their aggressiveness [3]. Eventually, most patients die because of refractory disease. Therefore, novel therapeutic approaches are urgently needed.

For many years, treatment regimens consisted of alkylating agents, anthracyclines and corticosteroids, and overall survival was poor (average three years) [4]. The introduction of bone marrow transplantation in the 1990s was a successful step in enhancing life expectancy. Landmarks in myeloma therapy in the last decade were the development of proteasome inhibitors such as bortezomib and carfilzomib as well as the introduction of immunomodulatory drugs such as thalidomide, lenalidomide, and pomalidomide, which are currently standard of care [5]. Recently, therapies including monoclonal antibodies against CD38 and B cell maturation antigen (BCMA) as well as CAR T cell therapies reveal promising results [6,7].

MM is a disease with high levels of both inter- and intra-patient heterogeneity [8,9]. At least seven subtypes have been identified corresponding to genetic lesions that are regarded as the initiating events in tumorigenesis. Beside genetic alterations, the tumor microenvironment plays a significant role in this disease [10]. Myeloma cells are mostly restricted to the bone marrow and heavily depend on cellular crosstalk. They modulate adjacent cells to support their growth and drug resistance via production of cytokines, chemokines, and other cofactors. These messengers trigger signaling cascades including NF-κB, JAK2/STAT3, Hedgehog, Notch, TGFβ-, and Wnt pathway [11]. Furthermore, molecules within the RAS/RAF/MEK/ERK as well as the PI3K/AKT-pathway are evolving as promising therapeutic targets [12].

Natural compounds often constitute the basis for the development of effective therapies. In the last four decades, about a quarter of all approved drugs were of natural origin (not including biologicals) and another quarter was inspired by nature, e.g., by using the molecules’ pharmacophore [13]. This is even more the case for anticancer drugs, where 25% of the approved compounds were of natural origin or nature-derived and 38% were mimicked by organic synthesis, meaning that drug discovery from nature is still the most common way for the development of novel therapeutics.

However, secondary (or specialized) plant metabolites do not only serve as potential lead structures for new drugs but in many cases are also part of our nutrition, not with respect to their caloric value but as accompanying substances with our intake of fruits and vegetables. The regular consumption of these substances might well play a role in the prevention of tumor development, given the fact that they are present in considerable concentrations [14]. Therefore, the present review not only aims to discuss antimyeloma plant natural products with respect to their eventual use as therapeutics or structural lead compounds, but also with regard to their dietary function.

## 2. Methods

A literature search was carried out for the time frame from 1945–2020 using the Web of Science citation indexing service. Thereby, the term “multiple myeloma” was used in combination with the words “natural product”, “natural compound”, “natural substance”, “naturally occurring”, or “plant-derived” resulting in 552 publications. Additionally, the term “multiple myeloma” was searched alone, and the results were reduced to the field of plant science, giving 36 hits. Duplicates were removed and the remaining publications were reviewed by title, abstract, and text resulting in 180 publications.

Literature data was evaluated for the use of appropriate cell lines and techniques ignoring studies of extracts without identifying single molecules. Moreover, only those compounds were included into this review, which were tested on at least one human myeloma cell line (also neglecting cell lines that are EBV-transformed B-lymphocytes). Thus, a total of 92 natural products with reported antimyeloma activity were obtained and divided into compound classes (alkaloids, phenolics, and terpenes) and, depending on the number of compounds, into several subclasses, thereby defining the structure of this review. Compound names (mostly trivial names) and configurations were taken “as is” from the original publications and names of the investigated plant species were checked using “The Plant List” [15].

The data discussed in this review is additionally summarized in two tables. Table 1 gives an overview of the investigated myeloma cell lines as well as the IC_50_ or EC_50_ values obtained from assays of the compounds’ antiproliferative effects or measurements for the inductions of apoptosis. Table 2 summarizes the investigated pathways and regulated molecules. Moreover, reported synergisms or inhibitions with other drugs used for the treatment of myeloma as well as eventual tests on primary myeloma cells are listed in this table. Additionally, applied tumor models and investigations of cells of the tumor-microenvironment are indicated.

## 3. Results and Discussion

### 3.1. Alkaloids and Other Heteroaliphatic Compounds

#### 3.1.1. Alkaloids

The first compound class discussed in this review are alkaloids. Alkaloids are well-known for their cytotoxic effects and are therefore used for the treatment of different cancers [16]. Most prominent examples are proto-alkaloids, such as the taxane derivatives paclitaxel and docetaxel, as well as the vinca alkaloids vinblastine, vincristine, vindesine, and vinorelbine, which belong to the group of indole alkaloids. With regard to multiple myeloma, the use of taxane derivatives has not been reported; however, vinca alkaloids have been components of myeloma therapy for a long time until more specific therapies were developed.

**Figure 1:**
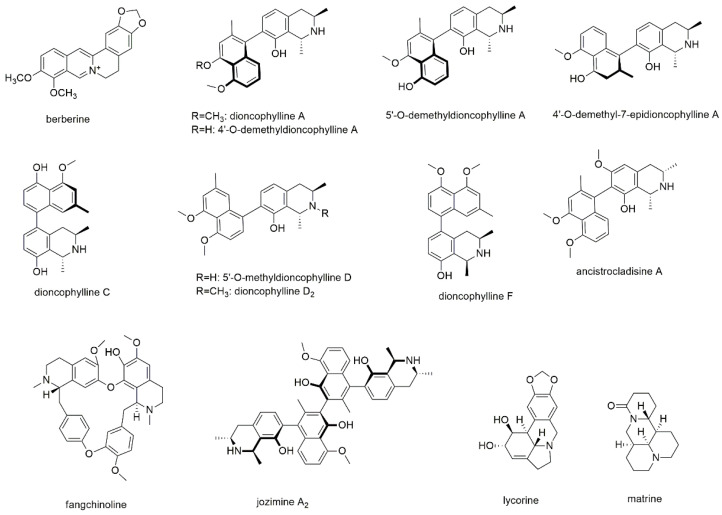
Alkaloids with reported activity against myeloma cell lines.

Our literature search revealed that most reports have been made for the group of isoquinoline alkaloids, with twelve components, and another two compounds of the phenanthridine type (Figure 1).

Three studies deal with the effect of berberine on myeloma cells applying RPMI8226 and U266 cell lines with miRNA screening methods [17,18,19]. Berberine, a well-known isoquinoline alkaloid of the protoberberine type, is distributed among the Berberidaceae, Papaveraceae, and Ranunculaceae plant families and mostly isolated from different *Berberis* species [20,21]. Berberine is known for its proapoptotic and autophagic potential against many types of cancer but also for its chemosensitizing properties suggesting its use in combination therapy [21,22,23]. With regard to MM, berberine downregulated miRNA-21 expression and significantly upregulated programmed cell death 4 (PDCD4) leading to apoptosis and G2 phase cell cycle arrest in RPMI8226 cells [17]. However, specific apoptosis was only 8% at a concentration of 75 µM and the IC_50_ for growth inhibition was 135 µM after 48 h of incubation. Therefore, the main effect is the regulation of miRNA-21 also known as “oncomir” and as such driving tumor growth. The authors, furthermore, hypothesize that the suppression of cell growth might (at least partly) result from the modulation of the IL-6/STAT3 pathway and that the increased expression of PDCD4 might result in the suppression of the p53 signaling pathway. In the following, Feng et al. systematically investigated common signaling pathways and found that three miRNA clusters (miR-99a~125b, miR-17~92, and miR-106~25) were significantly downregulated in MM cells after treatment with berberine [18]. As the three clusters are linked by TP53, ErbB, and MAPK, the authors suggest that these signaling pathways might be responsible for the suppression of cell growth. Additionally, the miR-99a~125b cluster is proposed as a potential target for the treatment of MM. Gu et al. came to similar results after investigating the miR-106b/25 cluster in berberine treated MM cells [19]. Here, inhibition of both p38 MAPK and phopho-p38 MAPK was detected, indicating the miR-106b/25 cluster functioning as oncogene.

Li et al. isolated several naphtylisoquinoline alkaloids from the Congolese liana *Ancistrocladus ileboensis*, which were tested for their apoptotic activity on INA6 cells using annexin-V-/PI staining and analysis by flow cytometry [24]. Of the isolated compounds, dioncophylline A, C, D_2_, and F, as well as 4′-O-demethyldioncophylline A, 4′-O-demethyl-7-epidioncophylline A, 5′-O-methyldioncophylline D, and ancistrocladisine A showed activities with EC_50_ values ranging from 0.22 to 32.0 µM after 72 h of incubation (Table 1). In the same study, 5′-O-demethyldioncophylline A, a related compound isolated from *Triphyophyllum peltatum* was tested showing an EC_50_ value of 1.5 µM. Except for dioncophyllines C and F, all tested compounds showed myeloma cell selectivity as compared to peripheral blood mononuclear cells (PBMCs). With regard to the structural properties, naphtyl coupling to position 7 of the isoquinoline ring, as, e.g., for dioncophylline A, is superior (for both activity and selectivity) than coupling to position 5, as observed for dioncophyllines C and F. Furthermore, N-methylation, as present in dioncophylline D_2_, decreases the activity compared to compounds with secondary amine functions. In contrast, the configurations of the two vicinal methyl groups seem to have a lesser impact on the compounds’ activity as well as the methylation pattern of the two distal hydroxyl-groups.

Fangchinoline, a bisbenzylisoquinoline alkaloid was investigated for its activity on U266 and the chronic myeloid leukemia cell line KBM5 using a variety of assays [Jung et al., 2019]. Fangchinolin is one of the two major alkaloids of *Stephania tetrandra* a traditional remedy in China and other oriental countries [25]. The compound attenuated the growth of the cell lines and lowered NF-κB as well as AP-1 activation. The authors determined attenuated phosphorylation of IκB kinase and p65 as possible mechanisms for the effects and furthermore observed a significant enhancement in TNFα-driven apoptosis.

Another dimeric alkaloid, the naphtylisoquinoline dimer jozimine A2 was isolated from *Ancistrocladus abbreviatus* and tested for its effect on MM1S and two other cancer cell lines using an MTT assay [26]. The IC_50_ value for MM1S was determined with 5.0 µM after 24 h of incubation.

The second class of alkaloids with reported antimyeloma activity are phenanthridine type alkaloids, namely lycorine and matrine. First isolated more than one hundred years ago, lycorine was subject of numerous pharmacological studies showing low toxicity and at the same time high potency against various cancer cell lines [27]. Therefore, the chemical structure of lycorine was used as natural lead for further drug development. Wang et al. investigated the effect of lycorine on myeloma cell lines ARP1, KMS11, ANBL6, and ANBL6 and found that the compound inhibited proliferation by decreasing ALDH1^+^ cells, which are supposed to be cancer stem cells [28]. Furthermore, lycorine was found to act via the Wnt/β-catenin pathway by lowering β-catenin protein levels and to exhibit synergistic effects when combined with bortezomib. Additional synergisms were described for combinations with pomalidomide, doxorubicin, and the alkylating agent melphalan, though the latter combination showed less pronounced effects. These results were as well observed in bortezomib resistant cells, indicating the compound’s potential to overcome bortezomib resistance and thus displaying a promising agent for MM treatment, either alone or in combination with other chemotherapeutics. In this study, lycorine was used up to a concentration of 5 µM.

The second phenanthridine type alkaloid is matrine, a bioactive ingredient of *Sophora flavescens* and *Sophora tonkinensis*, two medicinally used plant species in China and many Eastern countries [29,30,31]. The compound was found to possess a variety of pharmacological activities comprising different types of cancer [29,30,31,32]. Same as lycorine, also matrine was used as natural lead compound, also because it is very stable and shows only a few functional groups; therefore, it can be easily modified [31,32]. Investigations on the antimyeloma potential of matrine were conducted with RPMI8226 and U266 cell lines as well as bone marrow mononuclear cells using MTT assays [33]. Additionally, apoptotic cells were studied by Hoechst 33258 staining and flow cytometry and different mechanisms were evaluated by Western blotting. Activation of caspase-3 and poly (ADP-ribose) polymerase, upregulation of Bim expression and downregulation of bcl-2 and survivin expression, as well as inhibition of phosphorylated AKT were identified as responsible mechanisms for the observed effects. However, with IC_50_ values in the millimolar range, the therapeutic potential of the drug must be questioned.

#### 3.1.2. Other Heteroaliphatic Compounds

**Figure 2:**
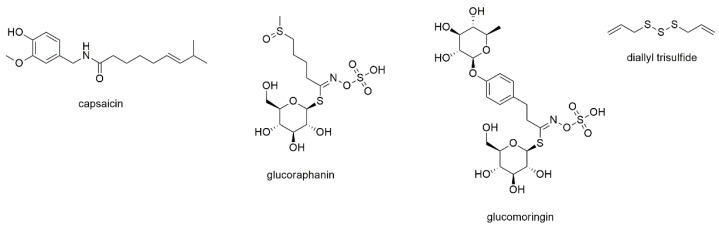
Heteroaliphatic compounds with reported activity against myeloma cell lines.

This section describes four compounds, which contain the heteroatoms nitrogen and/or sulfur within their structure (Figure 2).

The first compound is capsaicin, the pungent principle of chili pepper [34,35]. The compound belongs to the capsaicinoids, a compound class sometimes included within the alkaloid family [34]. However, as these compounds neither show a cyclic nitrogen nor basicity (the nitrogen is an amide and thus neutral), the compound does not fulfil the criteria for an alkaloid. Capsaicin has been repeatedly studied for its anticancer activity with most reports dealing with cancers of the liver and the intestine [36,37,38]. With regard to its effect on myeloma cells, the compound was found to inhibit constitutive and IL-6 induced STAT3 activation as well as activation of JAK 1 and c-Src [39]. Only little effects on STAT5 and no effect on ERK 1/2 were observed. Furthermore, capsaicin downregulated the expression of STAT3-regulated gene products and induced the accumulation of cells in G1phase. As the downregulation of STAT3 could be reversed by pervanadate, an inducer of cellular tyrosine phosphorylation, the authors suggest an involvement of a protein tyrosine phosphatase. Additional experiments revealed antiproliferative activity against MM1S and U266 cells and induction of apoptosis, with a potentiating effect of capsaicin in combination with bortezomib and thalidomide. Finally, capsaicin was administered i.p. to athymic mice, demonstrating inhibition of myeloma tumor growth also in vivo.

Brunelli et al. studied glucoraphanin and glucomoringin, two glucosinolates present in species of the Brassicaceae family, on various cancer cell lines [40]. RPMI8226 cells were used to study antiproliferative effects resulting in IC_50_ values of 7.7 and 6.9 µM, respectively, after 48 h of incubation. Both compounds inhibited NF-κB activity and induced apoptosis, with stronger effects observed for glucomoringin. Antimyeloma activity of glucomoringin was also observed in a SCID-mouse model, however, toxicity was discussed at prolonged (3 week) treatment.

Diallyl trisulfide is an organosulfur compound derived from garlic, a natural product that has been ethnomedicinally used in China for more than a thousand years [41,42]. It is one of the decomposition products of allicin, that rapidly occurs (besides diallyl sulfide and diallyl disulfide) after cutting or squeezing the fresh cloves [41,43]. Diallyl trisulfide was investigated on RPMI8226 and H929 cells using MTT assay, resulting in high micromolar IC_50_ values [42]. Further experiments were performed investigating the effects on the myeloma stem cell fraction defined as side population using Hoechst staining. The compound reduced the survival rate, blocked colony formation, induced cell cycle arrest and promoted apoptosis of SP cells to an extent comparable to bortezomib treatment.

Interestingly, all compounds of this subsection are food ingredients, meaning that depending on one’s preference for vegetables or spices, they are consumed on a regular basis. With three of the four components showing apoptotic activity and antiproliferative effects at low micromolar concentrations, they might well play a role in cancer chemoprevention provided that they are bioavailable and do not undergo rapid cooking degradation or metabolic elimination. Thermal decomposition particularly accounts for glucosinolates, which are significantly degraded upon boiling [44]. Thus, either non-thermal processing of cruciferous vegetables or their use as fresh or raw extracts is proposed to benefit from their effects [44,45]. Capsaicin, in contrast, does not suffer from thermal degradation but displays only low oral bioavailability as it is rapidly metabolized in the liver [34]. Therefore, current research focusses on the use of nanoparticles or other delivery systems in order to increase its therapeutic potential [35,36].

### 3.2. Phenolics

#### 3.2.1. Quinones

**Figure 3:**
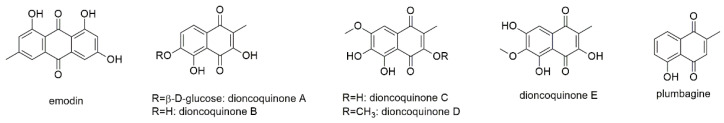
Quinones with reported activity against myeloma cell lines.

This section contains one anthraquinone, namely emodin, and six naphthoquinones (Figure 3).

Emodin, an active component from the Chinese traditional drug *Rheum palmatum*, exhibited activity against several cancers and showed synergistic and protective effects in combination with chemotherapy [46]. It was tested for its antimyeloma potential on RPMI8226, U266, and KMS12PE myeloma cell lines [47]. Lowest IC_50_ values were determined in RPMI8226 cells (37.7 µM after 24 h of incubation) and were somewhat higher for the other cell lines (Table 1). The authors revealed that emodin inhibited IL-6 induced activation of JAK2 and phosphorylation of STAT3, resulting in decreased expression of the myeloma survival protein myeloid cell leukemia 1 (Mcl-1). Emodin, furthermore, triggered activation of caspase-3 and caspase-9 without affecting the expression of other bcl-2 family members except Mcl-1. According to the authors, the selective inhibition of the JAK2/STAT3 pathway and downregulation of Mcl-1 suggests a good potential as anti-myeloma reagent. However, in a recent report of the European Food Safety Authority (EFSA), the compound (together with other hydroxyanthracene derivatives) was found to produce genotoxic effects in vitro and to increase the risk of colorectal cancer [48]. Though the genotoxic effect of emodin was only observed in vitro, *Aloe* extracts containing emodin were also found genotoxic in vivo. Based on these findings, the European Commission proposed to prohibit the use of hydroxyanthracene derivatives, and all preparations containing them, in food [49].

Bringmann et al. isolated dioncoquinones A and B from callus culture of the tropical liana *Triphyophyllum peltatum* [50]. Both compounds were measured for their apoptotic potential using INA6 and RPMI8226 cell lines, with EC_50_ values of 59 and 78 µM (dioncoquinone A) as well as 11 and 18 µM (dioncoquinone B), respectively. Both compounds did not affect healthy PBMCs. In a follow-up study, three more naphthoquinone derivatives (dioncoquinones C-E) were isolated and another already known natural product (plumbagin) was yielded by organic synthesis [51]. Dioncoquinone C showed similar activity against the viability of INA6 cells with an EC_50_ of 14 µM, whereas dioncoquinones D and E were less effective, with values of 80 and 100 µM, respectively. However, PBMCs were not affected by any of the three compounds. Plumbagin, instead, showed much higher inhibitory activity, with an EC_50_ of 0.8 µM, but also affected healthy PBMCs to the same extent. Comparison of dioncoquinone structures showed that the highest activity is achieved with free hydroxyl groups in position 3 and 6, as present in dioncoquinones B and C. Comparison with dioncophyllines isolated from the same plant species and also from *Ancistrocladus ileboensis*, revealed that dioncoquinones did not reach the activities of their alkaloid counterparts (see Section 3.1.1). Of the group of naphthoquinones, plumbagin was subjected to various studies confirming its anticancer effects and suggesting its further development as antineoplastic drug [52,53]. However, with regard to MM, the apoptotic effects of plumbagin were equally pronounced against healthy PBMCs.

#### 3.2.2. Phenylethanoids and Phenylpropanoids

**Figure 4:**
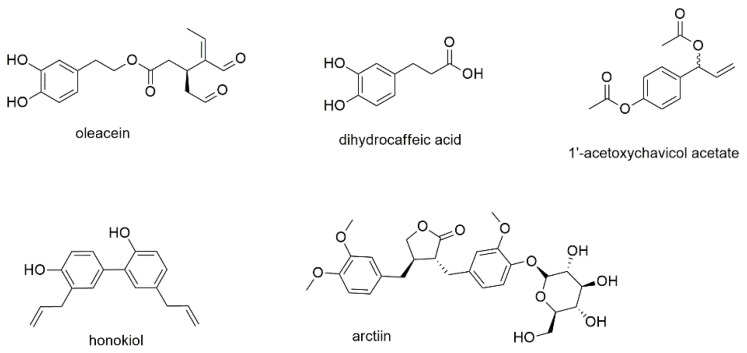
Phenylethanoids and phenylpropanoids with reported activity against myeloma cell lines.

This section contains one phenylethanoid, namely oleacein, two simple phenylpropanoids, and two condensed phenylpropanoids, also known as lignans (Figure 4).

Oleacein, a phenylethanoid ester of hydroxytyrosol with a secoiridoidic acid, is one of several active polyphenols found in olive oil [54]. The compound was tested for its effect on cell viability against eight different human myeloma cell lines and showed IC_50_ values in the range of 5 to 20 µM (Table 1). Viability was reduced even in the presence of bone marrow stromal cells, while healthy PBMCs were not affected. Accumulation of both acetylated histones and α-tubulin as well as the downregulation of several histone deacetylases were identified as mechanism of action. Oleacein, moreover, showed synergistic effects with carfilzomib, a proteasome inhibitor, indicating a potential use for combination therapy.

Dihydrocaffeic acid is one of the simplest phenylpropanoids and (together with other hydroxycinnamic acids) a substructure of numerous natural products. The compound itself was investigated (together with several other polyphenols) on U266 and RPMI8226 cell lines showing IC_50_ values of 61.9 and 344.0 µM, respectively [55]. However, the more interesting finding in this report was the detection of chemical interactions between some compounds with bortezomib. These interactions were only found for structures with vicinal aromatic hydroxyl groups that directly reacted with the boronic acid moiety of bortezomib thus antagonizing the effect of the proteasome inhibitor.

In two studies, the antimyeloma effect of 1′-acetoxychavicol acetate, a component of the ethnobotanically used plant species *Alpinia galanga,* was investigated [56,57]. The first study found that the compound acted via the NF-κB pathway, decreasing the nuclear location of NF-κB but increasing the accumulation of cytosolic NF-κB in RPMI8226 cells [56]. 1′-acetoxychavicol acetate also induced cleavage and thus activation of caspases 3, 8, and 9 and inhibited serine phosphorylation as well as degradation of IκBα. The effect was also studied in vivo, where treatment of NOD/SCID mice with 1′-acetoxychavicol acetate significantly decreased tumor growth. A follow-up study by the same group revealed that the compound upregulates the expression of both TNF-related apoptosis-inducing ligand/Apo2 ligand (TRAIL/Apo2L) and TRAIL receptor death receptor 5 (DR5) [57]. A third study investigated several analogues of 1′-acetoxychavicol acetate and found a more potent agent (TM-233) [58]. Thereby, the ethenyl feature of 1′-acetoxychavicol acetate was replaced by a 9-anthryl moiety, leading to increased induction of apoptosis compared to its parent compound.

Honokiol a lignan biphenol present in traditionally used *Magnolia* species was found to exhibit anticancer activity on various types of cancer, thereby modulating multiple oncogenetic targets [59,60]. The compound was, furthermore, found to enhance the antineoplastic effect of several chemotherapeutic agents, e.g., doxorubicin and paclitaxel, in combination treatment [61]. The effects of honokiol were also investigated in U266 myeloma cells [62]. Similar to the simple phenylpropanoid 1′-acetoxychavicol acetate, the dimeric phenylpropanoid was found to suppress NF-κB activation and to block TNF-induced phosphorylation, ubiquination, and degradation of IκBα. An in vivo mouse model confirmed the downregulation of NF-κB.

The last compound of this section, arctiin, is a classical lignan, where the two phenylpropane units are linked by the central carbons of their side chains [63]. It was first identified in *Arctium lappa*, a popular medicinal herb and health supplement for the anti-influenza treatment in East Asia [64]. Arctiin was investigated for its effect on MM1S, RPMI8226, and U266 cells. The authors describe effects in U266 cells, the only cell line with constitutive activation of STAT3, with an IC_50_ value below 20 µM after 24 h of incubation [65]. Further experiments revealed that arctiin abrogated the constitutive activation of Scr phosphorylation as well as JAKs 1 and 2. The compound, furthermore, enhanced mRNA and protein levels of protein tyrosine phosphatase ε (PTPε). However, usage of complete medium instead of starving medium abrogated the effects. Additionally, arctiin treatment failed to block IL-6 induced STAT3 phosphorylation in RPMI8226 and MM1S cells. The targeting of different pathways by honokiol compared to arctiin might be explained by their different geometries. Arctiin belongs to the dibenzylbutyrolacton type lignans and contains two stereocenters in the lactone ring, as well as a β-oriented glucose moiety, resulting in an almost ring-shaped geometry. Honokiol, in contrast, shows an S-form and, moreover, much lower polarity.

#### 3.2.3. Diarylheptanoids and Pyrones

**Figure 5:**
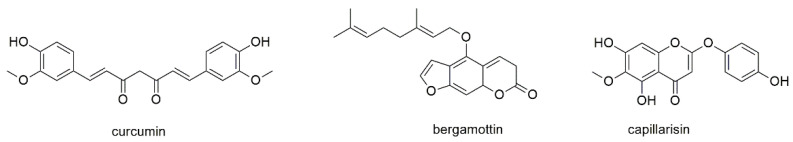
Diarylheptanoids and pyrones with reported activity against myeloma cell lines.

This section deals with the diarylheptanoid curcumin and with one α- and two γ-pyrones (Figure 5).

Curcumin is the major active component in turmeric (*Curcuma longa*), a commonly used spice and coloring food additive [66]. It is one of the most investigated natural products with regard to anticancer activity knowing to regulate miRNA expression and modifying a series of signaling pathways in various types of cancer (e.g., endometrial cancer or human glioblastoma) [67,68,69]. With regard to MM, curcumin has been investigated for its effect on U266, MM1S, MM1R, and RPMI8226 cell lines and completely suppressed cell proliferation at a concentration of 10 µM [70]. Curcumin was found to inhibit IFNα-induced STAT1 phosphorylation and IL-6 induced STAT3 phosphorylation, whereas phosphorylation of STAT5 was not affected. Additionally, the compound affected dexamethasone-resistant MM1R cells. In a more recent study, curcumin was compared to β-sesquiphellandrene (see Section 3.3.1), revealing IC_50_ values below 25 µM (U266) and 10 µM (MM1S) after 72 h of incubation [71]. Curcumin was also part of a clinical trial (NCT00113841) evaluating the clinical tolerance and safety of the compound in myeloma patients in a pilot study, thereby measuring the change of NF-kB protein expression in PBMCs. However, a consecutive phase II study (NCT01269203) on the reduction of symptoms during the treatment with lenalidomide was withdrawn. 

Bergamottin is a constituent of grapefruit juice and the only natural α-pyrone with reported antimyeloma activity, though its compound class is more commonly referred to as coumarin or furanocoumarin [72]. Bergamottin was found to be antineoplastic in different tumor models and also showed acceptable bioavailability [73]. In MM cells, the compound was found to abrogate constitutive STAT3 activation through inhibition of phosphorylation of JAK 1 and 2 as well as c-Src. Bergamottin, moreover, induced the expression of tyrosine phosphatase SHP-1, downregulated the expression of STAT3-regulated gene products, and significantly potentiated the apoptotic effects of bortezomib and thalidomide in U266 myeloma cells.

Capillarisin, a constituent of *Artemisia capillaris*, was also found to inhibit constitutive and inducible STAT3 activation through activation of upstream JAK 1 and 2 and c-Src [74]. Likewise, the potentiation of the apoptotic effect of bortezomib was also observed for capillarisin. However, with an IC_50_ of 200 µM (after 36 h of incubation) the compound does not seem promising for therapeutic use. 

Though displaying three different chemical structures, all three compounds in this section act via inhibition of the STAT3 (and STAT1) pathway. Similar to the group of heteroaliphatic compounds, also here two components are likely to be consumed regularly, given a favor for spicy food or grapefruit juice, respectively.

#### 3.2.4. Stilbenoids

**Figure 6:**
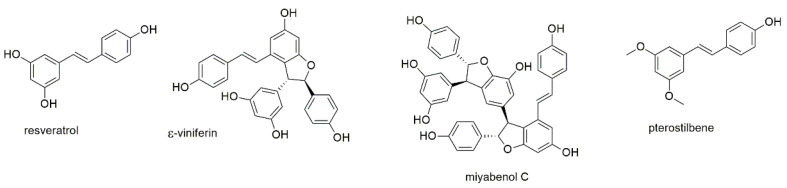
Stilbenoids with reported activity against myeloma cell lines.

This section discusses resveratrol, one of the most investigated natural products against multiple myeloma, and three of its derivatives, including one dimeric and one trimeric stilbenoid (Figure 6). 

Similar to curcumin, resveratrol is an anticancer compound of major interest and known to act via an array of different pathways [75]. It, moreover, shows immunomodulatory effects in the tumor microenvironment and has chemosensitizing properties, suggesting its potential use in therapy-refractory cancer [76,77]. Several studies investigated the antimyeloma properties of resveratrol [78,79,80,81,82,83], a compound present in red wine and linked to its health beneficial effects [84]. Boissy et al. investigated the effect of resveratrol on the growth of RPMI8226 and OPM2 myeloma cells and found induction of apoptosis [78]. In addition, they investigated the effect of resveratrol on cells of the tumor microenvironment. Thus, they show that the compound inhibited NF-κB ligand (RANKL)-induced formation of osteoclasts from primary monocytes. Furthermore, resveratrol promoted the expression of osteocalcin and osteopontin in human bone marrow mesenchymal stem cells and stimulated their response to 1,25(OH)_2_ vitamin D_3_. Together with the up-regulation of 1,25(OH)_2_ vitamin D_3_ receptor expression, resveratrol thus prevented osteoclast formation and promoted osteoblast differentiation. Sun et al., furthermore, revealed that resveratrol downregulated the expression of anti-apoptotic proteins (bcl-2, bcl-xL, and XIAP) and upregulated the expression of the proapoptotic protein Bax [79]. In addition, resveratrol inhibited the constitutive expression of matrix metalloproteinases MMP-2 and MMP-9 and suppressed their function in MM cells. MTT assays revealed inhibition of cell proliferation with IC_50_ values of 72, 74, and 80 µM against RPMI8226, U266, and KM3 cells, respectively, after 48 h of incubation. Invasion assays using the same cell lines resulted in IC_50_ values of 64, 93, and 153 µM, respectively. Bhardwaj et al. showed that resveratrol not only interfered with the NF-κB pathway, but also inhibited the constitutive and IL-6 induced activation of STAT3 [80]. The authors, moreover, discovered that resveratrol potentiated the anti-apoptotic activity of bortezomib and thalidomide. Li et al. evaluated synergistic effects of resveratrol with carfilzomib and found the combination to inhibit cell growth by promoting oxidative stress in MM cells [81]. In the last study, resveratrol was investigated for its effect on the overexpression of NEAT1 in U266 and LP1 cells [82]. NEAT is a long non-coding RNA found to promote ß-catenin expression and unfolded protein response (UPR), an essential survival mechanism for myeloma cells. The authors identified the overexpression of this RNA in a screen on patient tissues and found it also highly expressed in myeloma cell lines. Resveratrol reversed the effect of NEAT1 overexpression on MM cells through the Wnt/β-catenin signaling pathway and thus counteracted proliferation, migration, and invasion of MM cells. IC_50_ values for the two investigated cell lines were determined with 40.7 (LP1) and 33.7 µM (U266), respectively, after 72 h of incubation.

Barjot et al. investigated the antiproliferative and antiapoptotic effects of two naturally occurring resveratrol oligomers, namely ε-viniferol and miyabenol C, on RPMI8226 and U266 cell lines [83]. The oligomer ε-viniferol showed higher IC_50_ values on RPMI8226 than the resveratrol monomer (45.7 vs. 26.3 µM) and not statistically different IC_50_ values for U266 cells (30–40 µM). The trimer miyabenol C, however, was found to be more effective, with IC_50_ values of 20.8 and 12.1 µM, respectively. The latter compound was also the most effective in inducing cell death. All three components revealed potential to activate caspases and were associated with disruption of the mitochondrial membrane potential. 

Pterostilbene (or 3′,5′-dimethoxyresveratrol) is a compound found in blueberries and grapes [85]. Pterostilbene was investigated on H929 and bortezomib-resistant H929R cell lines, showing IC_50_ values of 22.83 and 34.80 µM, respectively, after 48 h of incubation. Mechanistically, the compound was found to cause apoptosis via a caspase-dependent pathway as well as through downregulation of p-AKT and activation of the MAPK signaling pathways. Pterostilbene, moreover, showed synergistic effects with histone deacetylase inhibitors panobinostat and vorinostat. Xie et al. investigated pterostilbene on additional cell lines ARP1, OCIMY5, and RPMI8226 with comparable IC_50_ values [86]. The compound caused cell cycle arrest at G0/G1 phase by enhancing ROS generation and reducing mitochondrial membrane potential (MMP). The authors thereupon hypothesized that the anti-tumor effect may be caused by the activation of the extracellular regulated protein kinases (ERK) 1/2 and c-Jun N-terminal kinase (JNK). The anti-myeloma effect of pterostilbene was also demonstrated in a mouse model, where the compound provoked a reduction of tumor volume after intraperitoneal injection. Reduction of tumor size in mouse models was, moreover, observed for DCZ0801, a semi-synthetic derivative obtained through fusion of pterostilbene with oxophenamide [87,88]. In the latter study, the analogue was found to suppress glycolysis via inactivation of the AKT/mTOR pathway [88].

The extensive studies on resveratrol (in contrast to many other compounds) revealed various mechanisms of actions, which were partly confirmed for its derivatives. Although the reported IC_50_ values of the above-described compounds do not suggest further promotion as potential chemotherapeutics, resveratrol was still evaluated in a phase II study for its safety and tolerability (NCT00920556). However, the study was terminated early due to minimal efficacy signals observed. Due to their anticancer properties and their presence in fruits, stilbenoids have attracted the interest of food industry promoting their health-beneficial effects [89,90]. With regard to their bioavailability, resveratrol is known to be rapidly degraded in the liver, but several of its metabolites show protective effects against cancer [91]. Less is known about its oligomers, which also raises the demand for proper toxicological evaluation of these compounds for their use in food industry [90]. Pterostilbene, in contrast, was found pharmacologically safe showing no organ-specific or systemic toxicity [92]. In addition, the compound is more lipophilic than resveratrol showing higher intestinal permeability and metabolic stability [93]. The higher bioavailability and the fewer adverse effects compared to resveratrol suggest pterostilbene as promising chemopreventive nutritional supplement [93,94]. 

#### 3.2.5. Chalcones

**Figure 7:**
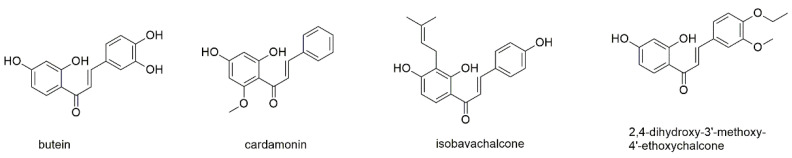
Chalcones with reported activity against myeloma cell lines.

Four chalcones will be reported in this section, which all show very close structural relations (Figure 7). 

The first chalcone to be reported is butein, a compound that is known from several plant species, such as Chinese and Tibetan traditional medicinal herbs *Caragana jubata* and *Rhus verniciflua* [95]. Butein shows several pharmacological properties, such as antioxidant, antihypertensive, anti-inflammatory, and anticancer activity [96,97]. The latter effects were found to result from the induction of apoptosis, cell cycle arrest, and the regulation of NF-κB related gene products [97]. In myeloma cells, the compound inhibited proliferation of U266 and MM1S cells with IC_50_ values of around 10 and 30–40 µM, respectively, after 72 h of incubation and inhibited both constitutive and IL-6 induced STAT3 activation [95]. The suppression was mediated through the inhibition of activation of JAK 1 and 2 as well as the upstream kinases c-Src. Butein, moreover, induced the expression of the tyrosine phosphatase SHP-1 and downregulated the expression of STAT3-regulated genes. Additionally, the compound showed synergistic apoptotic effects with both bortezomib and thalidomide. Interestingly, the vicinal aromatic hydroxyl groups of butein did not seem to antagonize the effect of bortezomib as was reported for several polyphenols [55], even though both compounds were applied together.

Cardamonin (2′,4′-dihydroxy-6′-methoxychalcone) is present in the traditional Chinese medicinal plant *Alpinia katsumadae,* which is meanwhile classified as *Alpinia haianensis*, and known to display anticancer properties via modulation of multiple signaling pathways, such as mTOR, NF-κB, AKT, STAT3, Wnt/β-catenin, and COX-2 [98,99]. The compound is lacking the two hydroxyl groups in ring B and shows an additional methoxy group in ring A instead, making it more lipophilic than butein. Cardamonin was tested for its antiproliferative effect on RPMI8226 and U266 showing IC_50_ values of approximately 60 and 45 µM, respectively, after 24 h of incubation as well as values of 10 and 15 µM after 48 h of incubation [98]. The apoptotic potential of the compound was determined with an EC_50_ of 50 µM after an incubation period of 24 h. Thereby, cardamonin activated caspase-3 and PARP and suppressed the expression of anti-apoptotic proteins of the bcl-2 family. Cardamonin, furthermore, inhibited NF-κB through suppression of IKK expression and IκBα phosphorylation and downregulated the expression of NF-κB-regulated gene products.

Isobavachalcone is a compound present in the seeds of *Psoralea corylifolia*, a traditional Chinese remedy [100]. The compound is of even lower polarity than the two before mentioned compounds showing a logP value of approximately 4 (and thus twice the value of butein), which mostly results from the prenyl moiety. Isobavachalcone showed antiproliferative effects against H929 cells with an IC_50_ value of approximately 10 µM after 48 h of incubation. The compound induced apoptosis but concomitantly enhanced protective upregulation of autophagy. Consequently, combined treatment with autophagy-inhibitors such as chloroquine enhanced cell death.

2,4-dihydroxy-3′-methoxy-4′-ethoxychalcone, a compound present in the traditional medicinal herb *Caragana pruinosa*, was investigated for its antiproliferative effect on RPMI8226, MM1S, and U266 cells [101]. With IC_50_ values of 26.0, 18.4, and 15.0 µM, respectively, after 24 h of incubation, the compound seems to be the most potent among the investigated chalcones. With regard to the involved mechanism, 2,4-dihydroxy-3′-methoxy-4′-ethoxychalcone was found to induce apoptosis via activation of caspases 3 and 9, upregulation of Bad and downregulation of bcl-2 and the survival pathway PI3K/AKT/mTOR. 

Though the four chalcones discussed in this section showed varying polarities, their antiproliferative potential does not seem to differ significantly. It is also comparable to the potential of the before discussed stilbenoids, as is their mechanism of action. However, in contrast to the stilbenoids, the chalcones presented in this review were all isolated from traditionally used medicinal plants rather than from fruits. 

#### 3.2.6. Flavonoids

**Figure 8:**
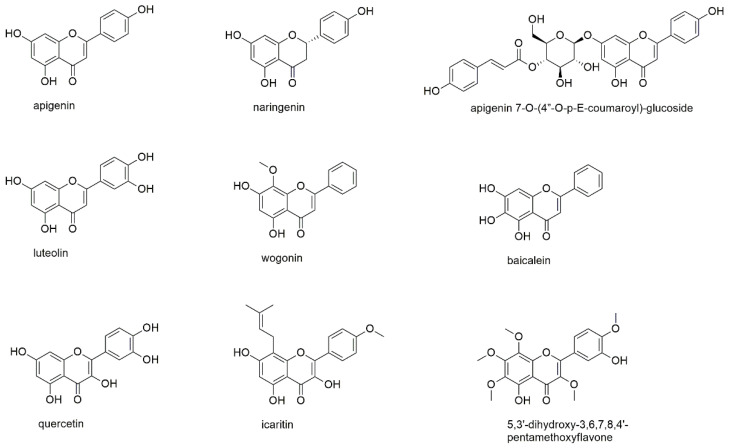
Flavonoids with reported activity against myeloma cell lines.

This section describes nine flavonoids, of which one is a flavanone, five are flavones and three are of the flavonol-type (Figure 8). 

Apigenin is one of the most abundant flavonoids in plant kingdom and therefore has been the target of various investigations for its use as anticancer agent and for its role in cancer prevention [102,103,104]. In these studies, the compound showed anticancer potential via induction of apoptosis and cell cycle arrest as well as the downregulation of matrix metalloproteinases and interaction with miRNAs. However, apigenin was also found to undergo rapid elimination, thus raising the need for, e.g., nanoformulations to increase its bioavailability [104]. The compound has been studied for its effect on U266 and RPMI8226 cell lines and primary MM cells, where it inhibited proliferation (without affecting healthy PBMCs), influenced cell cycle progression and induced programmed cell death [105]. Apigenin thereby blocked CK2 activity, leading to inactivation of multiple kinases such as the constitutive and inducible STAT3, AKT, ERK, and NF-κB as well as their upstream kinase partners. Treatment with apigenin also downregulated the expression of the antiapoptotic proteins Mcl-1, bcl-2, bcl-xL, XIAP, and survivin. Although the study on the ubiquitin-proteasome pathway by Wu and Fang was performed on solid cancer cell lines, the described effect on different catalytic activities of the proteasome (chymotrypsin-/trypsin-like) might as well play a role in multiple myeloma [106].

Naringenin is the 2,3-dihydro derivative of apigenin and thus belongs to the class of flavanones, which possess at least one stereocenter. However, in the case of naringenin racemization occurs in aqueous solutions [107]. Same as apigenin, naringenin showed anticancer activity via different mechanisms of action and additionally exhibited protective effects against natural and chemical toxic agents [108]. Additionally, for naringenin bioavailability is a major issue, which has been evaluated in several clinical trials [109]. Willer et al. isolated the compound from damiana (*Turnera diffusa*), a natural aphrodisiac used in Latin America [110]. Naringenin was investigated against H929 and U266 cells, showing significant cytotoxic effects at concentrations >25 µM in MM cells compared to healthy PBMCs. In the same study, apigenin 7-O-(4″-O-p-E-coumaroyl)-glucoside, a more complex apigenin derivative, was investigated. The compound, however, exhibited lower effects on both cancerous and healthy cells and was less selective for myeloma cells.

Luteolin, same as apigenin, is of high abundance in the plant kingdom and often shows co-occurrence. Likewise, it exhibits anticancer effects via different signaling pathways and against various cancer types, with the most promising results found in pancreatic and breast cancer models [111,112,113]. With regard to its antimyeloma activity, the only report found in literature was in the Chinese language [114]. However, from the English language abstract it could be deduced that the compound inhibited proliferation and caused apoptosis and autophagy in RPMI8226 cells. Luteolin was, furthermore, part of the abovementioned study on the ubiquitin-proteasome pathway showing comparable effects to apigenin [106]. The effect of luteolin on myeloma cells was confirmed in a recent study of our own group, where it showed even higher rates of cell-death induction than apigenin [115].

Wogonin, one of the major constituents of *Scutellaria baicalensis*, was investigated on RPMI8226 cells, showing apoptotic effects with decreased levels of anti-apoptotic bcl-2 protein and significantly increased levels of pro-apoptotic Bax protein [116]. Wogonin decreased phosphorylation of AKT at Ser473 and thus suppressed its activity. Moreover, the compound was found to fit well within the AKT1 ligand binding domain by molecular docking studies. Though the authors propose wogonin as a potential therapeutic agent, an IC_50_ value of 143.2 µM (after 24 h of incubation) does not corroborate this suggestion. However, since MM cells express AKT1 and AKT2 with the latter being even more important, the isoform selectivity of wogonin could explain the high IC_50_ values in this cell type.

Another compound present in *Scutellaria baicalensis*, but also in *Oroxylum indicum*, is baicalein [117,118]. Same as the abovementioned flavonoids, baicalein was found to be active against a variety of cancer cells and thereby acted via different pathways [119], however, also with limited oral bioavailability [120]. With regard to MM, baicalein was studied for its effect on U266 cells and showed an IC_50_ value of 60 µM after 24 h of incubation [121]. The compound was found to act via a cereblon-dependent down-regulation of the lymphoid transcription factors IKZF1 and IKFZ3. Cereblon is the primary target for the immunomodulatory drugs commonly used in myeloma therapy; thus, combination therapies could be promising and might ameliorate the low apoptotic potential observed when used as single agent. 

Quercetin is another highly abundant flavonoid in plant kingdom and the flavonol counterpart to luteolin, also displaying vicinal hydroxyl groups in ring B. This structural feature was one of the key findings of a study on polyphenols (see Section 3.2.2), where quercetin among other compounds was found to chemically interact with the boronic acid substructure of bortezomib [55]. IC_50_ values of quercetin against U266 and RPMI8226 cell lines were determined with 50.5 and 120.5 µM after 48 h of incubation. While no signaling pathways were investigated in this study, quercetin, same as other flavonoids, modulated a number of different pathways in other cancer models [122]. However, in prostate cancer, Bax detachment from bcl-xL and stimulation of caspases was the most appreciated route, and a combination with a TNF-related apoptosis-inducing ligand has been recommended to overcome resistance to apoptosis [123].

Icaritin is a prenylated flavonol from the ethnomedicinally used genus *Epimedium* and is currently tested in a phase III clinical trial for advanced hepatocellular carcinoma [124]. The compound also showed promising results in several hematological malignancies [125]. With regard to MM, icaritin was studied for its antiproliferative effect on U266 cell line with IC_50_ values of 36.6, 10.1, and 8.6 µM after 24, 48, and 72 h of incubation without affecting normal hematopoiesis [126]. Acting mainly via inhibition of IL-6/JAK2/STAT3 signaling, icaritin also showed in vivo activity in a xenograft mouse model suppressing tumor growth.

5,3′-dihydroxy-3,6,7,8,4′-pentamethoxyflavone is one of the components of *Gardenia obtusifolia*, a traditional Thai panacea [127]. The compound was investigated on various cancer cell lines including three human myeloma cell lines (U266, RPMI8226, and MM1S). Thereby, 5,3′-dihydroxy-3,6,7,8,4′-pentamethoxyflavone inhibited the proliferation of cancer cells to a concentration of 1 µM, however, after approximately 96 h of incubation. The compound was found to act through modulation of AKT-GSK3β pathways and induction of cyclin-dependent kinase (CDK) inhibitors. In a follow-up study, 5,3′-dihydroxy-3,6,7,8,4′-pentamethoxyflavone inhibited both constitutive and IL-6 inducible STAT3 activation in myeloma cells, leading to the suppression of proteins involved in proliferation, survival, and angiogenesis [128]. Additionally, the apoptotic effects of thalidomide and bortezomib were significantly potentiated by 5,3′-dihydroxy-3,6,7,8,4′-pentamethoxyflavone.

Flavonoids are widely distributed in plant kingdom fulfilling important roles in the plants’ physiological processes [129]. Due to their high abundance and the ready availability of the major flavonoids, they belong to the most studied compound subclasses. However, neither the major flavonoids (apigenin, luteolin, and quercetin) nor the more specific compounds discussed in this section exhibited remarkable antiproliferative effects. Here, icaritin was the most promising constituent showing an IC_50_ value in the low micromolar range and, furthermore, suppressing tumor growth in vivo. 

#### 3.2.7. Isoflavones and Xanthones

**Figure 9:**
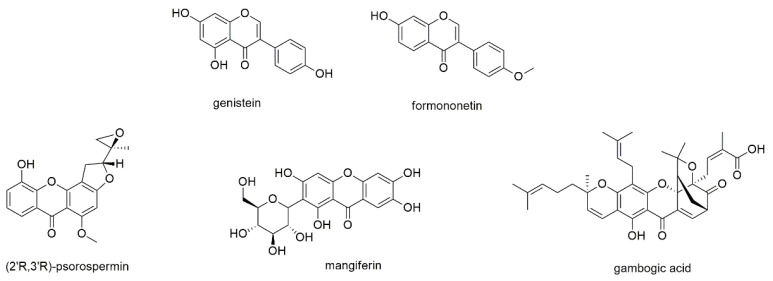
Isoflavones and xanthones with reported activity against myeloma cell lines.

This section describes two well-known isoflavones, namely genistein and formononetin, as well as three xanthone derivatives (Figure 9).

Isoflavones are a compound class not restricted to but mainly found in species of the Fabaceae family. One of the most studied compounds of this class is genistein, which displays the isoflavone counterpart of apigenin and is present in soy products as well as species of the genus *Sophora* [130,131]. It has been subject of numerous investigations with regard to its regular intake with soy products, and despite its low bioavailability was found beneficial in the prevention of, e.g., breast cancer [132,133,134]. The compound has also been investigated in two clinical trials, with promising results [135]. Li et al. investigated the antimyeloma effect of genistein on the proliferation of OPM2 and U266 cells and determined IC_50_ values of 46.7 and 128.8 µM, respectively, after 72 h of incubation [130]. Genistein enhanced cleavage and thus activation of caspases 3, 7, and 9, cleavage of PARP as well as downregulation of bcl-2 family members. He et al. studied the compound’s effect on RPMI8226 cells and found that NF-κB was downregulated and nuclear retention of p65 was prevented [136]. Genistein, moreover, downregulated the expression of NF-κB related gene products and suppressed constitutive AKT phosphorylation. The suppression of NF-κB and the modulation of caspase 3 activity of genistein were also observed by Xie et al., who studied its effect on U266 cells [131]. The authors, furthermore, revealed that the downregulation of NF-κB resulted from up-regulation of micro-RNA 29b.

Formononetin, another well-known isoflavone present in *Astragalus membranaceus*, *Trifolium pratense*, *Glycyrrhiza glabra*, and *Pueraria lobata*, was investigated on U266 and RPMI8226 cell lines [137]. The compound suppressed constitutive STAT3 and STAT5 as well as upstream kinases JAK1/2 and c-Src via an increased production of reactive oxygen species (ROS). Formononetin, moreover, downregulated the expression of STAT3-regulated anti-apoptotic, angiogenic, and proliferative gene products, which correlated with the induction of caspase-3 activation and the cleavage of PARP. The effect of formononetin was also investigated in vivo, where suppression of tumor growth in xenograft mice was observed after intraperitoneal injection.

Psorospermin is a xanthone present in the roots and stembark of the African plant *Psorospermum febrifugum*, where it occurs in the (2′R,3′R)-configuration [138]. Fellows et al. synthesized all four enantiomers of the compound and tested them for their effect on several cancer cell lines, including RPMI8226, RPMI8226S, RPMII8226/DOXIV, and RPMI8226/D40 cells. Of all four enantiomers, the naturally occurring (R,R)-enantiomer was the most potent with IC_50_ values of 0.072, 0.036, 0.097, and 0.037 µM, respectively, after 96 h of incubation. The pronounced effect against doxorubicin-resistant RPMI8226/D40 cells was further investigated by Carey et al., who found that the retention of doxorubicin was enhanced after pretreatment with psorospermin [139]. As overexpression of P-glycoprotein is the main reason for doxorubicin-resistance, the authors conclude that the effect is due to mdr1/P-glycoprotein inhibition. Even more so, as neither transcription of mdr1 nor translation of P-glycoprotein were downregulated after application of the compound.

Mangiferin, a C-glucosyl xanthone, was investigated by Takeda et al. for its effect on RPMI8226 cell line [140]. The compound, that is known from plants such as *Mangifera indica*, was found to inhibit nuclear translocation of NF-kB by decreasing the expression of phosphorylated NF-kB-inducing kinase (NIK), XIAP, survivin, and bcl-xL proteins. Mangiferin, furthermore, inhibited NF-kB activation by increasing the expression of IκB protein. Though mangiferin also showed promising results in various other cancers, it was not further promoted due to its limited bioavailability [141]. Thus, current research focuses on the development of appropriate drug delivery systems, such as mangiferin-integrated polymers.

Three studies investigated the antimyeloma potential of gambogic acid. Gambogic acid is a caged polyprenylated xanthone and the major active ingredient of gamboge secreted from *Garcinia hanburryi* [142]. It exhibited pronounced effects against an array of different cancers and was therefore attributed promising therapeutic potential [143]. However, poor aqueous solubility and biodistribution combined with multi-targeting capacity led to unavoidable systemic toxicity [144]. Therefore, numerous studies focused on the minimization of these side-effects by means of nanotechnology [142,144]. The potential of gambogic acid against MM was evaluated in three studies [145,146,147]. Yang et al. investigated the compound’s effect on the proliferation of RPMI8226 cells and determined an IC_50_ value of approximately 2.5 µM after 12 h of incubation [145]. The effect was due to the accumulation of ROS, leading to the activation of caspase 3, cleavage of PARP, and the downregulation of SIRT1. Pandey et al. investigated the effect of gambogic acid on MM1S, U266, RPMI8226, and H929 cell lines and found that the compound downregulated the expression of homing receptor CXCR4 by inhibiting NF-kB DNA binding [146]. The direct interaction with CXCR4 was predicted by docking studies and subsequently confirmed by a quantitative chromatin immunoprecipitation assay, where gambogic acid inhibited p65 binding as well as phosphorylation of AKT, p38, and Erk1/2. The compound, moreover, abrogated RANKL- and MM cell induced differentiation of macrophages to osteoclasts through IL-6 inhibition. Wang et al. studied the effects of gambogic acid on expression of HIF-1α and its downstream target VEGF in U266 cells, showing that hypoxia-activated pathways were suppressed by the inhibition of the PI3K⁄AKT⁄mTOR signaling pathway [147]. As a result, IC_50_ values under hypoxia were significantly lower (0.4–1.6 µM) than under normoxic conditions (2.8 µM, both after 8 h of incubation). The compound, furthermore, decreased tumor volumes in mice by anti-angiogenic activity.

Same as flavonoids, also isoflavones have been subject of numerous pharmacological studies, but due to their restriction to the Fabaceae family, their abundance is quite lower. Genistein and formononetin display two major isoflavones, which (like their flavonoid counterparts) cannot be regarded as compounds with remarkable antimyeloma activity. In contrast, the rather less abundant group of xanthones yielded two compounds with pronounced effects, namely psorospermin and gambogic acid. While the latter compound showed low micromolar IC_50_ values against RPMI8226 and U266 cells (after only 12 and 8 h of incubation), psorospermin was even inhibiting normal and doxorubicin-resistant RPMI8226 cells with IC_50_ values ranging from of 36 to 97 nM (even though the incubation time of 96 h was quite long). Thereby, it was shown that psorospermin was not only acting through the abovementioned P-glycoprotein inhibition but also through topoisomerase II-mediated alkylation by its side chain [139].

#### 3.2.8. Gallic Acid Derivatives

**Figure 10:**
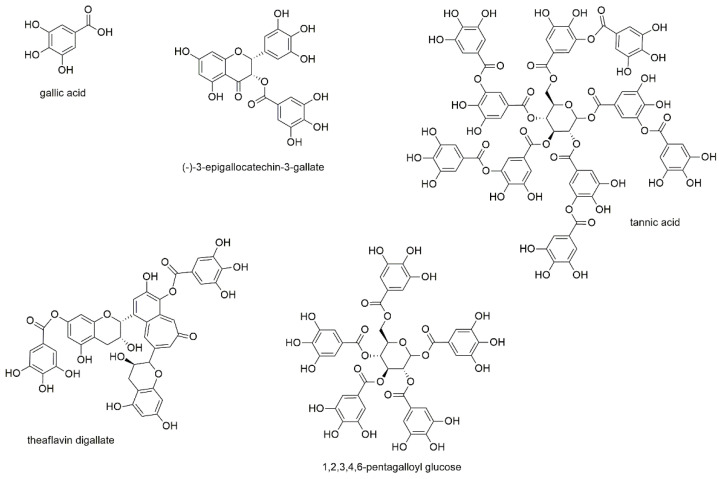
Gallic acid derivatives with reported activity against myeloma cell lines.

This section deals with gallic acid and four of its derivatives, of which two are galloylcatechins and two are galloylglucoses (Figure 10). 

Gallic acid, also known as 3,4,5-trihydroxybenzoic acid, as well as (-)-3-epigallocatechin-3-gallate and tannic acid were investigated for their effect on U266 and RPMI8226 cell lines [55]. IC_50_ values were determined with 23.3 and 96.8 µM for gallic acid, 28.0 and 58.8 µM for epigallocatechin gallate, as well as 12.5 and 11.0 µM for tannic acid. In the same study, the chemical interaction of vicinal aromatic hydroxyl groups with bortezomib was discovered (as already mentioned above), for which all three compounds show the necessary structural feature. Epigallocatechin gallate was furthermore the subject of a clinical trial evaluating blood counts in patients with monoclonal gammopathy of undetermined significance (MGUS) and/or smoldering MM (SMM) after treatment with epigallocatechin gallate-rich green tea extracts (NCT00942422). Both, MGUS as a premalignant stage of myeloma and SMM, where the patients are still lacking end-organ damage, are usually covered by a “watch and wait” therapeutic strategy. However, the outcome of the study and the statistical significance of the obtained results were low, also because of the small number of only eight enrolled patients. With regard to general anticancer activity, epigallocatechin gallate is by far the most investigated of these three compounds and has been attributed both preventive as well as therapeutic potential in the treatment of various types of cancer [148,149,150,151,152]. 

A screening for inducers of endoplasmatic reticulum stress in ARP1 and KMS11 myeloma cells yielded 97 out of 2000 marketed natural products [153]. Of these 97 compounds theaflavin digallate was chosen for further validation, during which IC_50_ values of 0.59 µM (ARP1) and 0.27 µM (KMS11) were determined.

Tseeleesuren et al. investigated pentagalloyl glucose (1,2,3,4,6-penta-O-galloyl-beta-D-glucopyranoside), a polyphenol found in numerous herbs, for its effect on RPMI8226, U266, and H929 cells [154]. IC_50_ values for the three cell lines were determined with 23.9, 36.2, and 10.2 µM, respectively, after 72 h of incubation. Thereby, the compound induced caspase 3 activity, and decreased MYC expression, a proto-oncogene that is frequently hyperactivated in MM. Additionally, pentagalloyl glucose lowered mRNA levels and reversed the mRNA expression of MYC target genes such as p21, p27, and cyclin D2. Additionally, in this study, the effect of bortezomib was antagonized in combination treatment, as has already been described for other polyphenols [55].

Though all five compounds of this subsection display tanning agents, they differ not only by their molecular weights but also by the type of gallic acid derivatives, with two compounds showing a central glucose molecule and two others a flavan-type scaffold. Of the latter type is theaflavin digallate, which consists of two condensed flavanols with two attached gallic acids, and by far shows the highest antiproliferative potential. Still, the type of derivative does not seem to be the sole responsible criteria, which becomes evident by the only moderate activity of epigallocatechin gallate, the second flavan-type derivative.

### 3.3. Terpenes

#### 3.3.1. Sesquiterpenes

**Figure 11:**
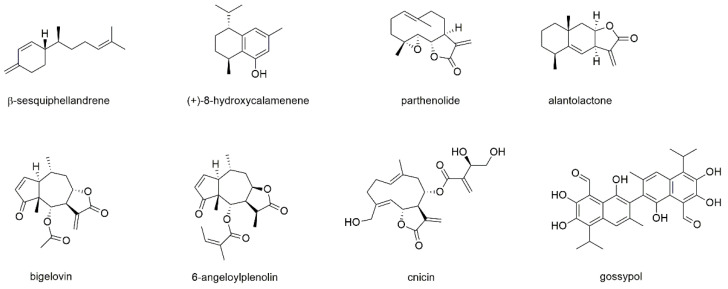
Sesquiterpenes with reported activity against myeloma cell lines.

Seven sesquiterpene monomers, of which five belong to the subclass of sesquiterpene lactones, and one sesquiterpene dimer will be discussed in this section (Figure 11). 

β-sesquiphellandrene was isolated from turmeric (*Curcuma longa*) together with five other compounds (α-curcumene, ar-, α-, β-, and γ-turmerone) [71]. All compounds were investigated for their cytotoxic potential on different cancer cell lines, including U266 and MM1S cells, and compared to curcumin. Thereby, β-sesquiphellandrene was the only active compound with IC_50_ values between 5 and 10 µM, which was equal to the effect of curcumin. In various other cancer cell lines (e.g., lung, colon) the compound was found to induce cytochrome C release, to activate caspases, and to induce PARP cleavage. β-sesquiphellandrene also downregulated cell survival proteins such cFLIP, bcl-xL, bcl-2, c-IAP1, and survivin, while the lack of NF-κB-p65 protein had no effect on the activity. Additionally, the compound was found to be synergistic with the myeloma drugs bortezomib and thalidomide. 

(+)-8-hydroxycalamenene, a compound present in the traditional Korean medicinal plant *Reynoutria elliptica*, was studied on U266 cell lines [155]. The compound blocked constitutive STAT3 activation through the inhibition of upstream kinases JAK1/2 and c-Src. (+)-8-hydroxycalamenene, moreover, inhibited the expression of gene products involved in the counteraction of apoptosis (bcl-2 and bcl-xL), proliferation (cyclin D1), and invasion (MMP-9) and potentiated the apoptotic effect of bortezomib. 

Parthenolide is a sesquiterpenoid and known NF-κB inhibitor present in the feverfew plant (*Tanacetum parthenium*) [156]. It contains an α-methylene-γ-lactone ring and an epoxide group that are able to interact with nucleophilic sites of biological molecules. Apart from the covalent reaction, parthenolide was found to modulate microtubule dynamics by interfering with the detyrosination of α-tubulin [157]. The compound showed pronounced effects against several cancer cell lines and seems to be a promising candidate for multimodal therapies [156,157]. MM inhibitory activity of parthenolide was tested on MM1S, MM1R, H929, U266, RPMI8226, and RPMI8226/Dox-resistant cells, showing IC_50_ values between 1 and 3 µM on all cell lines after 72 h of incubation [158]. The compound blocked IL-6 secretion from bone marrow stromal cells, rapidly induced caspase activation and cleavage of PARP, MCL-1, XIAP, and BID. Parthenolide, moreover, showed additive and synergistic effects when combined with dexamethasone and TNF-related apoptosis-inducing ligand, respectively. Gunn et al. investigated the compound’s effect on the apoptosis of RPMI8226 and U266 cells and determined EC_50_ values of around 25 µM after 18 h of incubation [159]. Parthenolide was also studied for its effect on MM-cancer stem cells (MM-CSC), which were selected from the cell lines via the potential to form colonies in methylcellulose. Thereby, MM-CSC showed preferential cytotoxicity compared to non-CSC myeloma cells. Addition of the bone marrow stromal compartment did not abrogate the effect. Parthenolide was investigated in a phase I dose escalation trial of feverfew extract in patients with cancer [160]. Thereby, doses equivalent to 1, 2, 3, and 4 mg parthenolide were applied as tablets and plasma levels of the compound were measured using liquid chromatography coupled to mass spectrometry. In this study, no significant toxicity was observed; however, no relevant plasma concentrations (>0.5 ng/mL) of parthenolide could be detected.

Alantolactone, which is present in the root of the medicinal plant *Inula helenium* was subject of numerous anticancer studies and found to affect different signaling pathways, namely p38, STAT3, NF-κB, and AKT [161]. The effect on myeloma cells was tested on OPM2, MM1S, MM1R, U266, H929, RPMI8226, and RPMI8226/BTZ-resistant cell lines, with IC_50_ values between 3 and 6 µM after 48 h of incubation [162]. The compound inhibited cell growth in the presence or absence of bone marrow-derived stromal cell line (HS-5) by caspase-3 activation and down-modulation of the activation of ERK 1/2. Alantolactone, furthermore, reduced the secretion of MM survival and growth-related cytokines and inhibited cytokine-induced osteoclastogenesis. 

Bigelovin, a sesquiterpene lactone present in the traditional Chinese medicinal plants *Inula britannica* and *Inula helianthusaquatilis*, was studied on U266, RPMI8226, MM1S, and MM1R cells where it exhibited antiproliferative effects with IC_50_ values between 0.5 and 0.99 µM after 24 h of incubation [163]. Mechanistically, the compound caused cell cycle arrest and inducted apoptosis by proteolysis of E2F1, which was overexpressed in 25–57% of MM patients investigated.

6-O-angeloylplenolin, a sesquiterpene lactone isolated from *Centipeda minima*, was tested for its apoptotic effect on MM1S, U266, and RPMI8226 cells, showing EC_50_ values of approximately 7.5 µM after 48 h of incubation. The effect was associated with caspase-3 and PARP cleavage [164].

Cnicin, which is present in species of the genus *Centaurea* [165], was investigated for its antiproliferative effect on RPMI8226, U266, H929, OPM2, LP1, MM1S, and MM1R cells [166]. The compound showed IC_50_ values between 3 and 13 µM after 48 h of incubation. Thereby, cnicin acted via downregulation of Pim-2, a serine/threonine kinase that is highly expressed in malignant but not in normal plasma cells. The cytotoxic effect was accompanied by activation of caspases, accumulation of reactive oxygen species and downregulation of NF-κB and was confirmed in co-culture with stroma cells as well as in an ex vivo chicken chorioallantoic membrane assay. Additionally, the combination of cnicin with myeloma drugs (melphalan, bortezomib, AKT-inhibitor) led to enhanced cell death.

Gossypol, the last compound of this section, is usually extracted from cotton plants [167,168,169]. The compound was first used as male contraceptive but was subsequently studied for its potential use against cancer, where it mainly targets bcl-2 family proteins [169]. Though gossypol appears like (and ultimately ends up as) a polyphenolic compound, biosynthetically it is a dimeric sesquiterpenoid and therefore discussed in this section [170]. Lin et al. studied the effect of gossypol on U266 cells and determined IC_50_ values of 9.0, 2.4, and 0.9 µM after 24, 48, and 72 h of incubation [167]. The compound induced apoptosis via activation of caspase-3 and caspase-9 as well as decreased expression of bcl-2 and bcl-xL. In vivo studies in BALB/c mice showed that growth inhibition of about 30% was obtained after administration of gossypol. Sadahira et al. investigated the compound’s behavior against U266 cells and showed that beside caspase-3 activation also cytochrome c release from mitochondria was induced [168]. Further studies showed that gossypol suppressed IL-6 signals, indicated by the inhibition of JAK2-, STAT3-, ERK1/2-, and p38-phosphorylation. The effect of gossypol was found to depend on the displacement of BH3-only proteins from bcl-2 and on the inhibition of IL-6 signaling, ultimately cumulating in bcl-2 dephosphorylation and Mcl-1 downregulation.

Of the eight investigated sesquiterpenes, seven compounds showed antiproliferative effects in the micromolar range or even below, such as bigelovin or also gossypol after 72 h of incubation. Though the effect of sesquiterpenes is often explained by the presence of an alkylating exocylic methylene group, this structural feature cannot display the sole criteria, as it is lacking in half of the effective compounds described in this section.

#### 3.3.2. Diterpenes

**Figure 12:**
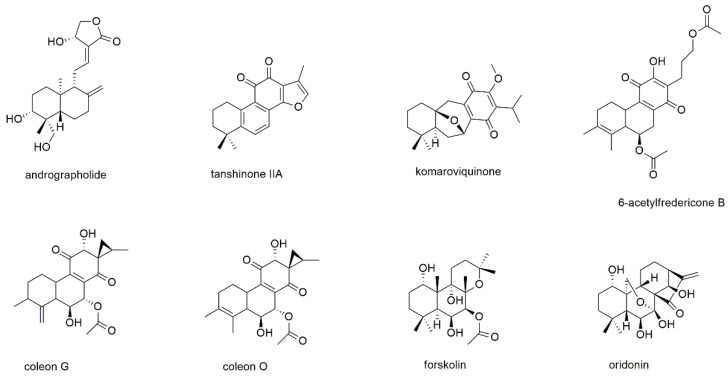
Diterpenes with reported activity against myeloma cell lines.

This section deals with eight diterpenoids of which five show quinone or quinone-like substructures (Figure 12). 

Andrographolide is a diterpene lactone isolated from *Andrographis paniculata* and inhibits the development of different types of cancer by regulation of Wnt/β-catenin, mTOR, VEGF-mediated intracellular signaling and TRAIL-mediated apoptosis [171,172]. Its MM inhibitory properties were investigated together with the sesquiterpene lactone parthenolide [159]. In this study, which was discussed in the section above, andrographolide induced apoptosis of both RPMI8226 and U266 cells with the same efficacy as parthenolide (EC_50_ values of 25 µM after 18 h of incubation). The compound also showed preferential toxicity toward MM-CSCs over non-tumorigenic MM cells. However, in contrast to parthenolide, the addition of the bone marrow stromal compartment abrogated andrographolide activity.

Tanshinone II_A_, a diterpenoid with an *ortho*-quinone substructure, is one of the bioactives from the traditional Chinese medicinal herb *Salviae miltiorrhiza* and known to display anticancer activity [173]. The compound was investigated for its antimyeloma effect on U266 cells, where it significantly increased the expression of microtubule-associated protein light chain 3 (LC3) II and thus induced autophagic cell death [174]. In KBM5 leukemia cells, tanshinone II_A_, augmented the phosphorylation of AMPK and attenuated the phosphorylation of mTOR and p70 S6K. The compound also dramatically activated the ERK signaling pathway including Raf, ERK, and p90 RSK in both dose- and time-dependent manner.

Komariviquinone is a compound present in *Dracocephalum komarovi*, which (same as the genus *Salvia*) belongs to the Lamiaceae family [175]. In contrast to the abovementioned tanshinone II_A_, komariviquinone shows *para*-quinone substructure. The compound was studied for its effect on the proliferation of myeloma patient-derived MUM24 cells, which were inhibited with an IC_50_ value of 0.65 µM after 48 h of incubation. 

The diterpene quinone 6-acetylfredericone B and the two-quinone like diterpenoids coleon G and O were tested on MM-CSC and RPMI226 cells [176]. The three compounds, which were isolated from *Plectranthus scutellarioides,* showed IC_50_ values of 17.6, 37.4, and 9.2 µM, respectively, against MM-CSC cells as well as values of 21.6, 38.4, and 8.4 µM, respectively, against RPMI8226 cells. The compounds were also evaluated for their ability to inhibit NF-κB, which was determined with IC_50_ values of 11.2, 11.0, and 9.7 µM, respectively. 

Forskolin is a labdane type diterpenoid present in the roots of the Indian plant *Coleus forskohlii*, which has been used for centuries in Hindu Ayurvedic medicine [177]. It is known to raise intracellular cAMP levels and has been found active against a variety of cancers. Forskolin has been tested for apoptosis induction in myeloid U266, H929, RPMI8226, OPM2, and INA6 cells, with EC_50_ values of approximately 80 µM for H929 and RPMI8226 cells as well as values of around 4 µM for U266, OPM2, and INA6 cells [178]. However, the main objective of this study was the investigation of therapeutic synergism of the cAMP-elevator forskolin and melphalan, cyclophosphamide, doxorubicin, bortezomib, and dexamethasone. It was shown that forskolin potentiated the killing induced by all the tested agents. Thereby, the combination with dexamethasone was found to be at least partly mediated by the proapoptotic bcl-2 family member BIM. 

Oridonin, a highly oxidated diterpenoid of the kauran-type, was isolated from *Isodon rubescens,* also referred to as *Rabdosia rubescens* [179,180,181]. The compound showed anti-angiogenic and antimetastatic properties against, e.g., breast, pancreatic, lung, colon, and skin cancer and was subsequently used as natural lead. Thereby, semi-synthetic approaches were focusing on modifications of the A-ring of the molecule or esterification of the hydroxyl group in position 14 [181]. With regard to MM, oridonin was investigated for its effect on the proliferation of RPMI8226 and RPMI8226/BTZ-resistant cells [179]. IC_50_ values were determined with 7.1 and 229.5 nM, respectively, after 48 h of incubation and were in the range of bortezomib (7.3 and 231.9 nM, respectively). Mechanistically, the compound sensitized MM cells via the PTEN/PI3K/AKT pathway, activating the expression of PTEN, a negative regulator of the PI3K/AKT pathway, while inhibiting the expression of p-AKT.

Not only sesquiterpenes, but also the subclass of diterpenes do provide a range of interesting compounds, among which komariviquinone and oridonin are certainly the most interesting candidates. Both compounds exhibited antiproliferative effects at submicromolar concentrations, whereby oridonin even showed a low nanomolar IC_50_ value against the normal RPMI8226 cell line and was even efficiently inhibiting BTZ-resistant RPMI8226 cells.

#### 3.3.3. Triterpenes

**Figure 13:**
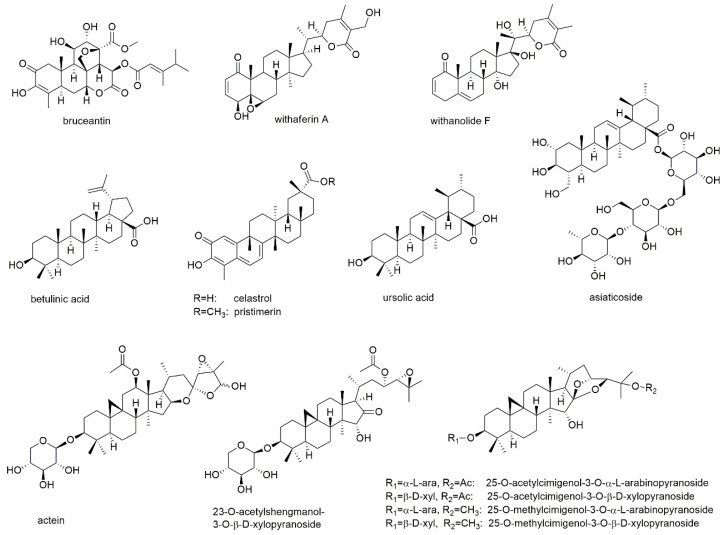
Triterpenes with reported activity against myeloma cell lines.

This section discusses 14 triterpenoids, of which seven compounds display triterpene glycosides (Figure 13). 

Bruceantin, a quassinoid present in different *Brucea* species, was investigated on MM and MM-CSC cells [182,183]. Quassinoids are exclusive for the Simaroubaceae family and are bitter tasting constituents, which biogenetically can be regarded as degraded triterpenoids [184]. Cuendet et al. studied the effect of bruceantin on the apoptosis of RPMI8226, U266, and H929 cells, determining EC_50_ values of 13, 49, and 115 nM, respectively [182]. Interestingly, the compound strongly downregulated c-MYC in the RPMI8226 cell line, but not in U266 or in H929 cells. Apoptosis resulted from proteolytic processing of procaspases and degradation of PARP and involved the mitochondrial pathway. The apoptotic effect of bruceantin on RPMI8226 cells was, furthermore, studied in a mouse xenograft model, where the compound led to significant regression of tumors. A study of bruceantin on MM-CSC cells found that the compound inhibited cell proliferation with an IC_50_ of 77 nM [183]. Moreover, bruceantin induced cell cycle arrest and apoptosis in MM-CSCs starting at a concentration of 25 nM. The compound also inhibited cell migration and angiogenesis presumably via the Notch pathway.

Two more triterpene lactones, withaferin A and withanolide F, were isolated from the leaves of *Withania adpressa*, a Moroccan endemic species and local remedy for the treatment of food intoxication [185]. Both compounds were found to inhibit proliferation of RPMI8226 and MM-CSC cells, with IC_50_ values of 0.2 and 0.3 µM (withaferin A) and 0.1 and 5.3 µM (withanolide F), respectively, after 72 h of incubation. Both compounds also inhibited NF-κB activity with IC_50_ values of 0.05 and 1.2 µM, respectively.

Betulinic acid, a pentacyclic triterpene of the lupane-type, was first isolated from *Ziziphus mauritiana*, which is meanwhile classified as *Ziziphus jujuba*, but is known to be present in various other plants, e.g., species of the birch tree (*Betula*) [186,187,188]. Though betulinic acid was found to possess some anticancer activity, it was rather suggested for combination therapies or as subject for further chemical modification, such as the semi-synthesis of more potent triphenylphosphonium derivatives [188,189]. The compound was studied for its antimyeloma effect on U266 and MM1S cell lines, where it inhibited constitutive activation of STAT3, c-Src kinase, JAK1 and JAK2 at a concentration of 50 µM [186]. Betulinic acid downregulated the expression of STAT3-regulated gene products such as bcl-xL, bcl-2, cyclin D1, and survivin, and induced the expression of the protein tyrosine phosphatase SHP-1. The compound’s antiproliferative effect was determined with IC_50_ values of 20 µM against U266 cells and 30 µM against MM1s cells, both after 72 h of incubation. Betulinic acid, additionally, enhanced the apoptosis in myeloma cells induced by thalidomide and bortezomib. Shen et al. investigated the effect of betulinic acid on U266 and RPMI8226 cells and found that the compound mediated cytotoxicity through induction of apoptosis (with an EC_50_ value of approximately 40 µM after 12 h of incubation), S-phase arrest, mitochondrial membrane potential collapse, and overwhelming ROS accumulation [187]. ROS overproduction directly resulted from inhibition of the NF-κB pathway, the latter mechanism was also demonstrated in a mouse xenograft model. Another xenograft model showed that betulinic acid initiated different types of cell death and that PPA2 was acting as a switch to regulate between apoptosis and autophagy [190].

Celastrol is an oleanane type triterpenoid, which has been isolated from the Chinese medicinal plant *Tripterygium wilfordii* (Thunder of God) about four decades ago [191]. It has been extensively studied for its anticancer activity [192,193,194], not only from mechanistic point of view but also to increase its potency through semi-synthetical modifications or the use of drug delivery systems [195,196]. Celastrol was studied for the growth inhibition of U266 cells, showing an IC_50_ value of 0.5 µM after 24 h of incubation [197]. It induced apoptosis of myeloma cells via activation of caspase-3 and blocking of NF-κB pathway. A study on the inhibition of c-Myc-Max heterodimers revealed celastrol as an inhibitor of the c-Myc protein, which is overexpressed in human cancers including myeloma [198]. However, due to the reactive quinone methide substructure of the compound, the inhibition did not result from prevention of dimerization but from directly altering the quaternary structure of the pre-formed dimer. Thus, the inhibition of c-Myc was less specific compared to triterpenoids lacking the quinone methide feature, but more potent. 

An earlier study investigated the effect of pristimerin, which is the methyl ester of celastrol and present in several species of the Celastraceae and Hippocrateaceae families [199]. The compound exhibited antiproliferative effects against a variety of cancer cell lines, with IC_50_ values in the low and sub-micromolar range. Pristimerin, which was detected in a chemical library search, was tested for its antiproliferative effect on various myeloma cell lines showing sub-micromolar IC_50_ values after 72 h of incubation (Table 1) [200]. Moreover, pristimerin was found to act via inhibition of phosphorylation of IκB, thereby stabilizing the inactive IκB/NF-κB complex as well as by causing an unfolded protein response in tumor cells and thus markedly suppressing NF-κB activity and cyclin D expression. Inhibiting both, proteasome chymotrypsin-like activity and NF-κB, pristimerin induced selective apoptosis with greater potency than other antineoplastic triterpenoids providing the rationale for pharmaceutical development of triterpenoid dual-function proteasome/NF-κB inhibitors. However, the abovementioned reactivity of celastrol (and the thus resulting unwanted pharmacological effects) also accounts for pristimerin, which shows exactly the same quinone methide substructure [198]. 

Ursolic acid, an ursane type triterpenoid, has been reported from numerous plant species and has been found to exert anticancer activity via mitochondria-dependent pathways [201,202]. Same as betulinic acid, the compound was studied for possible chemical or nanotechnological modifications in order to enhance its therapeutic effects [201,203]. With regard to MM, ursolic acid was studied for its effect on U266, MM1S, and RPMI8226 cells [204]. The compound inhibited constitutive and IL-6 induced STAT3 activation through the inhibition of activation of upstream kinases c-Src, JNK1/2, and ERK1/2. Ursolic acid, furthermore, downregulated the expression of STAT3-regulated gene products such as cyclin D1, bcl-2, bcl-xL, survivin, and Mcl-1, and induced the expression of tyrosine phosphatase SHP-1. Additionally, the compound potentiated the apoptotic effects of thalidomide and bortezomib. A more recent study found that ursolic acid interacted with deubiquinating protease USP7 in RPMI8226 cells, showing an IC_50_ value of 7 µM [205]. The antiproliferative effect was determined with an IC_50_ of 26.5 µM after 24 h of incubation. 

Asiaticoside is an ursolic acid derivative, showing additional hydroxylation, demethylation and foremost a trisaccharidic moiety, which is attached to the carboxyl group. The compound was tested on KM3/BTZ-resistant cells, where it exhibited an IC_50_ value of 12 µM after 48 h of incubation [206]. Same as ursolic acid, asiaticoside was found to act via modulation of the STAT3 pathway. Additionally, an increase of ROS levels as well as an upsurge in the expression of LC3-II was observed, which accompanied autophagic cell death induced by the compound. 

Six more triterpene glycosides with antimyeloma properties were detected in black cohosh, a traditional remedy for the alleviation of mild climacteric complaints [207]. Due to the complex metabolite pattern of black cohosh triterpenoids, three representative compounds for the respective substructures were initially tested on H929, OPM2, and U266 cells, uncovering the cimigenol type as the most active substructure. Subsequent evaluation of the apoptotic effect of four cimigenol derivatives showed EC_50_ values from 20 µM upwards after 24 h of incubation, however, with only slight selectivity compared to healthy PBMCs.

The subclass of triterpenes yielded several compounds with submicromolar IC_50_ values, three of them displaying a lactone ring (bruceantin, whitaferin A, and whitanolid F). This structural feature often also occurs in sesquiterpenes, which are then named sesquiterpene lactones. However, the latter group of compounds usually shows a pentacyclic lactone ring in contrast to the six-membered lactone ring present in the mentioned triterpenes. Out of these, the quassinoid bruceantin revealed the highest antiproliferative potential, with an even low nanomolar IC_50_ value against RPMI8226 cells. Additionally, celastrol and pristimerin, exhibited good inhibitory effects, as demonstrated on a variety of cell lines for the latter compound. Both, celastrol as well as pristimerin possess a quinone methide substructure and thus not only act via NF-κB but also show proteasome-inhibiting properties. However, this structural feature may also lead to unwanted pharmacological side effects, such as the formation of Michael adducts.

#### 3.3.4. Other Terpenes

**Figure 14:**
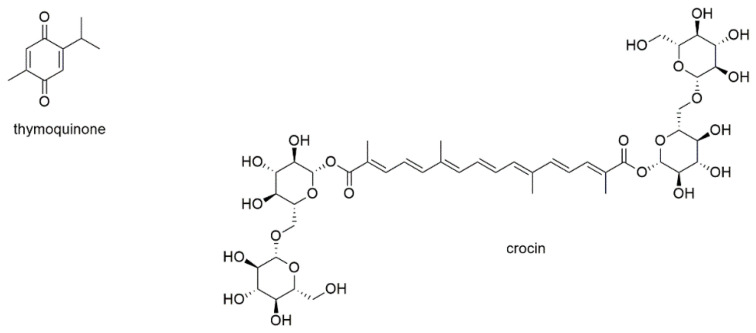
Mono- and tetraterpenes with reported activity against myeloma cell lines.

This section comprises the monoterpene thymoquinone and crocin, the only tetraterpenoid with reported antimyeloma activity (Figure 14). 

Thymoquinone is a major bioactive constituent in the volatile oil of black seed (*Nigella sativa*) and the only monoterpene with reported antimyeloma activity [208]. It has been subject of repeated studies evaluating its chemopreventive and anticancer effects [209]. Thereby, thymoquinone was targeting a variety of kinases and additionally showed effectiveness in murine tumor models [210,211]. In order to evaluate its antimyeloma potential, the compound was investigated for its antiproliferative effect on U266 and RPMI8226 cells, with IC_50_ values below 10 µM after 12 h of incubation [208]. Thymoquinone inhibited both constitutive and IL-6-inducible STAT3 phosphorylation, which correlated with the inhibition of c-Src and JAK2. Moreover, the compound was found to induce the expression of Src homology-2 phosphatase 2 that correlated with suppression of STAT3 activation and resulted in the downregulation of the expression of STAT3-regulated gene products, such as cyclin D1, bcl-2, bcl-xL, survivin, Mcl-1, and VEGF. In addition, the apoptotic effect of thalidomide and bortezomib was potentiated in combination treatment with thymoquinone.

The tetraterpenoid crocin is one of the main active principles in saffron (*Crocus sativus*) [212]. Due to the use of saffron as spice, the compound was as well subjected to several studies investigating its anticancer and chemopreventive effects [212,213]. With regard to MM, crocin was studied for its effect on U266 cells and effectively suppressed constitutive STAT3 activation through the inhibition of activation of protein tyrosine kinases JAK1, JAK2, and c-Src [214]. The compound furthermore suppressed the translocation of STAT3 to the nucleus and induced the expression of tyrosine protein phosphatase SHP-1. Crocin also downregulated the expression of STAT3-mediated gene products including bcl-2, BAX, CXCR4, VEGF, and cyclin D1.

Though both compounds derive from different compound subclasses, namely mono- and tetraterpenes, they do act via inhibition of the STAT3 pathway demonstrating how various chemical structures can still show similar mechanisms of action, as observed repeatedly during this review.

## 4. Summary and Conclusions

A total of 92 compounds with reported activity on at least one human myeloma cell line were found in literature (until the end of 2020) and discussed in this review. Thereby, each of the three main compound classes (alkaloids, phenolics, terpenes) yielded one or a few interesting compounds, which deserve special attention. For the class of alkaloids, the naphtylisoquinolin dioncophyllin A is pointed out, which induced apoptosis with an EC_50_ in the nanomolar range without showing significant toxicity. In addition, some of its natural derivatives did exhibit similar effects in the low micromolar range. From a chemical point of view, dioncophyllines are stable (with only a few compounds showing racemization) and thus can be produced by chemical synthesis, as demonstrated by Li et al. [24].

The two phenolic compounds theaflavin digallate and psorospermin similarly demonstrated antiproliferative potential in the nanomolar range, the latter compound affecting doxorubicin-resistant cells to comparable extent [138]. Additionally, of interest is the fact, that of four possible stereoisomers, the naturally occurring (R,R)-form was the most active, demonstrating the importance of correct stereochemical attribution when dealing with natural products as well as the stereoselectivity of pharmacological effects. Another xanthone derivative, namely gambogic acid is also worth mentioning. Although the compound’s antiproliferative effects were in the micromolar range (at least under normoxic conditions), it effectively suppressed hypoxia-activated pathways which might be important in vivo [147]. Consequently, gambogic acid was subject of additional studies evaluating its efficacy in combination therapies with bortezomib and other chemotherapeutics [142,215,216]. With regard to clinical trials, a phase IIa study comparing the efficacy and safety of different dosage schedules of gambogic acid in patients with advanced malignant tumors was reported [217]. Although no significant difference in the objective response rate (which was defined as primary endpoint) could be calculated, the greater disease control rates in the verum group suggest a favorable safety profile for gambogic acid at a dose of 45 mg/m². The class of terpenoids shows a range of interesting constituents, such as the highly oxygenated diterpenoid oridonin, which exhibited antiproliferative effects equal to those of bortezomib on RPMI8226 cells [179]. The quassinoid bruceantin as well showed low nanomolar IC_50_ values in RPMI8226 cells and inhibited MM-CSC in a nanomolar range [182,183], a fact that was also observed for withaferin A and withanolide F [185]. All three compounds are lactones, but also share the structural feature of a cyclohexenone ring, which might be a crucial functional group for the compounds’ activity. This hypothesis is corroborated by comparing the structures of celastrol and pristimerin, which also exhibited noteworthy effects [197,198,200]. Both compounds contain a structural analogue of the cyclohexenone ring, namely a quinone methide substructure, and showed proteasome as well as NF-κB inhibiting properties.

Thus far, four review articles dealing with MM and natural products were published [218,219,220,221]. Issa et al. focused on MM-CSCs and the most relevant stem cell characteristics such as resistance, self-renewal, differentiation, and migration [218]. Thereby, the key cellular signaling pathways were discussed and 17 natural products targeting hematological CSCs were presented. Kang et al. displayed different mechanisms by which natural products are known to exhibit their effects, e.g., induction of apoptosis, cell cycle arrest, anti-angiogenesis, and regulation of miRNAs [219]. For each mechanism, a set of active phytochemicals is highlighted, altogether discussing 23 single compounds and several plant extracts. Pojero et al. focused on natural polyphenols and presented a total of 18 components, which were discussed with regard to their biological effects and the affected signaling pathways [220]. In addition, the chemical synthesis of most of the compounds was depicted. The most recent review on natural products with MM inhibitory activity was written from a clinical point of view, listing possible therapeutic effects and conducted clinical trials for 23 single components and a few extracts [221]. Although the title of the review suggests dealing with dietary agents for MM chemoprevention and treatment, many of the discussed compounds rather derive from medicinal than edible plant species. Even though all four reviews reveal interesting findings, they all show only a selection of natural products with MM inhibitory activity. Thus, the present review article is the first one summarizing all plant natural products with demonstrated activity against human myeloma cell lines.

However, looking at the collected data it becomes evident that the reported literature derives from two different research areas, comprising phytochemical studies on the one hand and pharmacological investigations on the other hand. Thus, compounds with good initial results in testing proliferation and viability (e.g., dioncophylline A) are often not further investigated, whereas other constituents with only moderate activity are subjected to repeated pharmacological assays; alas, not seldom including unnecessary in vivo studies. This situation is likely due to the limited number of isolated substances available from phytochemical studies, which then restrict the execution to only one or a few convenient assays that were already used for the evaluation of fractions during the isolation procedure. In contrast, many pharmacological studies are conducted with commercially available substances, which do not have to be isolated from natural sources but can be readily ordered. Thus, the provision of larger amounts of substance is feasible (and affordable) allowing extensive investigations. Here, stronger co-operation between phytochemists and pharmacologists is desirable to eventually include the rare “hits” into subsequent pharmacological studies. Another option would be the collaboration with medicinal/organic chemists if the substances can be prepared synthetically. 

The many studies on well-known natural compounds, however, are still important. Firstly, the more detailed characterization of pathways contributes essentially to the understanding of the compounds’ mechanism of action, which usually also applies for related derivatives (as observed in this review). Secondly, as mentioned in the introduction, natural products still lay the basis for the majority of novel anticancer therapeutics and several anticancer drugs currently on the market or in clinical trials are of plant origin [13,222]. Especially, semi-synthetic derivatives continuously reveal potent anticancer drugs and therefore are of ongoing interest and subject of recent studies and reports [223,224]. Another fact that becomes evident when looking at the literature on anticancer drugs is that various compound classes afford potent candidates [225], which was also a finding of the present review. Thirdly, many of the investigated compounds are part of our nutrition, such as the heteroaliphatic and polyphenolic compounds discussed here. Knowledge on the mode of action for these substances may help to elucidate cancer chemopreventive properties, which are often postulated for dietary natural products [14,221]. This accounts especially for polyphenols, which are among the most studied compounds. However, many polyphenolic compounds, such as flavonoids and tannins, undergo rapid metabolization (e.g., glucuronidation) and degradation in the human gut [226,227]. Therefore, direct relation of eventual chemoprotective effects of these compounds with in vitro or in vivo results is difficult and especially depending on the route of administration. In addition, not only polyphenolics experience metabolic changes in vivo, but so do other compounds, as observed for a dose escalation trial of parthenolide, where no detectable amounts of the compound were found in human plasma after oral administration of up to 4 mg [160].

From the cell biological point of view, the investigations on pathways in these studies appear to be mostly conducted along the ones already known to have an impact on myeloma cells. For several studies, readouts are limited to effects on proliferation and viability and therefore a profound summary or classification is not feasible. 

Certainly: killing the bulk of myeloma cells, eradicating the myeloma stem cells and reconstituting a cancer-hostile microenvironment is a task that is hardly accomplished by a single compound. Natural products have attracted attention due to their large number of biological activities, fewer side effects, and high safety aspects. Utilizing recently designed culture setups (e.g., 3D co-cultures, humanized mouse models) and taking advantage of the development of sophisticated methods and approaches (e.g., proteomics, single cell sequencing) that enable complex experimental readouts, as well as the increasing availability of “real world” expression data from patients’ samples, will essentially contribute to further the success of natural compounds in anticancer research. 

## Figures and Tables

**Table 1:** Overview on reported measurements for inhibition of cell proliferation and for induction of apoptosis**.** Major cell lines are listed together with IC_50_ values for antiproliferative effects and the time of incubation (in parenthesis) or EC_50_ values for the induction of apoptosis, the latter indicated in italics. Additional cell lines are listed in the second to last column with the respective IC_50_ or EC_50_ as well as the time of incubation (both in parenthesis). All concentrations are given in µmol/L. The last column indicates the section of this review, in which the compounds are discussed in detail.

Compound Name	H929	INA6	MM1S	OPM2	RPMI8226	U266	Additional Myeloma Cell Lines	Section
1′-acetoxychavicol acetate					x	x		3.2.2
25-O-acetylcimigenol-3-O-α-L-arabinopyranoside	*27.3 (24 h)*			*>50 (24 h)*		*37.3 (24 h)*		3.3.3
25-O-acetylcimigenol-3-O-β-D-xylopyranoside	*30.6 (24 h)*			*39.7 (24 h)*		*>50 (24 h)*		3.3.3
6-acetylfredericone B					21.6 (24 h)			3.3.2
23-O-acetylshengmanol-3-O-β-D-xylopyranoside	x			x		x		3.3.3
actein	x			x		x		3.3.3
Alantolactone	4.13 (48 h)		3.85 (48 h) 3.19 (48 h)	3.56 (48 h)	4.32 (48 h)	5.79 (48 h)	RPMI8226/BTZ7 (5.03, 48 h) RPMI8226/BTZ100 (5.29, 48 h)	3.3.1
Ancistrocladisine A		*4.8 (72 h)*						3.1.1
Andrographolide					*~25 (18 h)*	*~25 (18 h)*		3.3.2
6-angeloylplenolin			*~7.5 (48 h)*		*~7.5 (48 h)*	*~7.5 (48 h)*		3.3.1
Apigenin					x	x		3.2.6
Apigenin 7-O-(4″-O-p-E-coumaroyl)-glucoside	x					x		3.2.6
Arctiin			x		x	<20 (24 h)		3.2.2
Asiaticoside							KM3 (12, 48 h)	3.3.3
Baicalein						~60 (24 h)		3.2.6
Berberine					135 (48 h)	x		3.1.1
Bergamottin			x			x		3.2.3
Betulinic acid			~30 (72 h)		x	*~40 (12 h)* ~20 (72 h)		3.3.3
Bigelovin			0.5–0.99 (24 h)		0.5–0.99 (24 h)	0.5–0.99 (24 h)	MM1R (0.5–0.99, 24 h)	3.3.1
Bruceantin	*0.115 (24 h)*				*0.013 (24 h)*	*0.049 (24 h)*		3.3.3
Butein			30–40 (72 h)			~10 (72 h)		3.2.5
Capillarisin						200 (36 h)		3.2.3
Capsaicin			x			5 (72 h)		3.1.2
Cardamonin					~60 (24 h) ~10 (48 h)	~45 (24 h) 15 (48 h)		3.2.5
Celastrol						0.47 (24 h)		3.3.3
Cnicin	~1 (72 h)		1–3 (72 h)	1–3 (72 h)	1–3 (72 h)	~3.5 (72 h)	LP1 (1–3, 72 h) MM1R (1–3, 72 h)	3.3.1
Coleon G					38.4 (24 h)			3.3.2
Coleon O					8.4 (24 h)			3.3.2
Crocin						x		3.3.4
Curcumin			5–10 (72 h)		x	10–25 (72 h)	MM1R	3.2.3
4′-O-demethyldioncophylline A		*2.7 (72 h)*						3.1.1
5′-O-demethyldioncophylline A		*1.5 (72 h)*						3.1.1
4′-O-demethyl-7-epi-dioncophylline A		*7.5 (72 h)*						3.1.1
Diallyl trisulfide	378.8 (24 h) 251.1 (48 h) 130.9 (72 h)				265.7 (24 h) 204.9 (48 h) 100.5 (72 h)			3.1.2
Dihydrocaffeic acid					344.0 (48 h)	61.9 (48 h)		3.2.2
2,4-dihydroxy-3′-methoxy-4′-ethoxychalcone			18.36 (24 h)		25.97 (24 h)	15.02 (24 h)		3.2.5
5,3′-dihydroxy-3,6,7,8,4′-pentamethoxyflavone			~50 (48 h)		~50 (72 h)	~50 (48 h)		3.2.6
Dioncophylline A		*0.22 (72 h)*						3.1.1
Dioncophylline C		*16.0 (72 h)*						3.1.1
Dioncophylline D_2_		*32.0 (72 h)*						3.1.1
Dioncophylline F		*21.0 (72 h)*						3.1.1
Dioncoquinone A		*29 (72 h)*			*58 (72 h)*			3.2.1
Dioncoquinone B		*11 /72 h)*			*18 (72 h)*			3.2.1
Dioncoquinone C		*14 (72 h)*						3.2.1
Dioncoquinone D		*80 (72 h)*						3.2.1
Dioncoquinone E		*100 (72 h)*						3.2.1
Emodin					37.7 (24 h)	x	KMS12PE	3.2.1
Epigallocatechin gallate					58.8 (48 h)	28.0 (48 h)		3.2.8
Fangchinolin						*>30 (24 h)*		3.1.1
Formononetin					~75 (72 h)	x		3.2.7
Forskolin	*~80 (72 h)*	*~4 (72 h)*		*~4 (72 h)*	*~80 (72 h)*	*~4 (72 h)*		3.3.2
Gallic acid					96.8 (48 h)	23.3 (48 h)		3.2.8
Gambogic acid	x		x		~2.5 (12 h)	~2.8 (8 h)		3.2.7
Genistein				46.76 (72 h)	x	128.82 (72 h)		3.2.7
Glucomoringin					6.08 (48 h)			3.1.2
Glucoraphanin					7.73 (48 h)			3.1.2
Gossypol				x		9.9 (24 h) 2.4 (48 h) 0.9 (72 h)		3.3.1
Honokiol						x		3.2.2
8-hydroxycalamene						*80 (24 h)*		3.3.1
Icaritin						36.63 (24 h) 10.05 (48 h) 8.60 (72 h)		3.2.6
Isobavachalcone	~10 (48 h)							3.2.5
Jozimine A_2_			5.0 (24 h)					3.1.1
Komaroviquinone							MUM24 (0.65, 48 h)	3.3.2
Luteolin					x			3.2.6
Lycorine							ANBL6, ANBL6BR, ARP1, KMS11	3.1.1
Mangiferin					x			3.2.7
Matrine					9059 (24 h) 6603 (48 h)	8777 (24 h) 6361 (48 h)		3.1.1
25-O-methylcimigenol-3-O-α-L-arabinopyranoside	*23.2 (24 h)*			*33.4 (24 h)*		*25.4 (24 h)*		3.3.3
25-O-methylcimigenol-3-O-β-D-xylopyranoside	*22.4 (24 h)*			*32,6 (24 h)*		*49.0 (24 h)*		3.3.3
5′-O-methyldioncophylline D		*2.6 (72 h)*						3.1.1
Miyabenol C					20.8 (24 h)	12.1 (24 h)		3.2.4
Naringenin	x					x		3.2.6
Oleacein	5–20 (48 h)		5–20 (48 h)	5–20 (48 h)	5–20 (48 h)	5–20 (48 h)	AMO1, AMOBZB, JJN3	3.2.2
Oridonin					0.0071 (48 h)		RPMI8226R (0.2295, 48 h)	3.3.2
Parthenolide	1–3 (72 h)		1–3 (72 h)		*~25 (18 h)*1–3 (72 h)	*~25 (18 h)*1–3 (72 h)	MM1R (1–3, 72 h) RPMI8226/Dox6 (1–3, 72 h)	3.3.1
Pentagalloyl glucose	10.24 (72 h)				23.92 (72 h)		U266B1 (36.18, 72 h)	3.2.8
Plumbagin		*0.8 (72 h)*						3.2.1
Pristimerin	0.15–0.3 (72 h)		0.15–0.3 (72 h)	0.15–0.3 (72 h)		0.15–0.3 (72 h)	KMS11 (0.75, 72 h) KMS18 (0.4, 72 h) MM1R (0.15–0.3, 72 h) OCIMY5 (0.15–0.3, 72 h) RPMI8226S (0.15–0.3, 72 h) SKMM2 (0.15–0.3, 72 h) UMTC2 (0.15–0.3, 72 h)	3.3.3
Psorospermin					0.072 (96 h)		RPMI8226S (0.036, 96 h)RPMI8226/Dox1V (0.097, 96 h)RPMI8226/Dox40 (0.037, 96 h)	3.2.7
Pterostilbene	22.83 (48 h)15.37 (72 h)				23.58 (72 h)		ARP1 (26.15, 72 h)H929R (34.8, 48 h)OCIMY5 (0.15–0.3, 72 h)	3.2.4
Quercetin					120.5 (48 h)	50.5 (48 h)		3.2.6
Resveratrol			~100 (24 h)		26.3 (24 h) 72 (48 h)	39.6 (24 h) 74 (48 h) 33.74 (72 h)	KM3 (80, 48 h) LP1 (40.72, 72 h) MM1R RPMI8226/Dox6 RPMI8226/LR5	3.2.4
β-sesquiphellandrene			5–10 (72 h)			5–10 (72 h)		3.3.1
Tannic acid					11.0 (48 h)	12.5 (48 h)		3.2.8
Tanshinone II_A_						x		3.3.2
Theaflavin digallate							ARP1 (0.59, 72 h), KMS11 (0.27, 72 h)	3.2.8
Thymoquinone					10 (24 h)	10 (48 h)		3.3.4
Ursolic acid			~25 (96 h)		26.56 (24 h)	~25 (96 h)		3.3.3
ε-viniferin					45.7 (24 h)	30.8 (72 h)		3.2.4
Withaferin A					0.17 (72 h)			3.3.3
Withanolide F					0.1 (72 h)			3.3.3
Wogonin					143.2 (24 h)			3.2.6

**Table 2:** Overview on investigated pathways and regulated molecules. Data collection is restricted to data presented in figures of the respective study dealing with investigations on myeloma cells only (MMP, mitochondrial membrane potential). Synergism and inhibition (marked with *) with other drugs are outlined (BTZ/bortezomib, Thal/thalidomide, Mel/melphalan, Dex/dexamethasone, Dox/doxorubicin, Cyclophos/cyclophosphamide, Pom/pomalidomide). Where applicable, the testing of the compounds on primary myeloma cells (CD138+ selected and patients’ bone marrow mononuclear cells stained for analysis of myeloma cells with CD38^++^/CD45^+/−^) is noted. Some compounds were also tested in tumor models (mostly mice, with one exception using chorioallantoic membrane (CAM) assay). MM-cancer stem cells (MM-CSC) are rather part of the malignant cells; however, here they are outlined in the section of tumor-microenvironment (Tumor-ME) to underline the importance of these tumor-initiating cells. Data in this section also includes data obtained using stroma cells and osteoclasts (BM, bone marrow).

Compound Name	Pathways Investigated	Regulated Genes/Proteins	Synergism/* Inhibition	Primary MM Cells Tested	Tumor Models	Tumor-ME Included	Section
1′-acetoxychavicol acetate	NFkB	NFkB, IkB	MG132, * Fas Ab,* PMA		NOD/SCID mice		3.2.2
1′-acetoxychavicol acetate	apoptosis	TRAIL					3.2.2
25-O-acetylcimigenol-3-O-α-L-arabinopyranoside	apoptosis	AnnV					3.3.3
25-O-acetylcimigenol-3-O-β-D-xylopyranoside	apoptosis	AnnV					3.3.3
6-acetylfredericone B	proliferation					MM-CSC	3.3.2
23-O-acetylshengmanol-3-O-β-D-xylopyranoside	apoptosis	AnnV					3.3.3
actein	apoptosis	AnnV					3.3.3
Alantolactone	cell cycle, apoptosis	CDK4,2 cyclin D1 E2,		stroma cells			3.3.1
Ancistrocladisine A	apoptosis	AnnV					3.1.1
Andrographolide	colony forming assay					MM-CSC, BM stroma	3.3.2
6-angeloylplenolin	apoptosis	PARP, Caspase 3, AnnV		CD138+			3.3.1
Apigenin	STAT3,AKT,NF-kB	CK2a, MCL-1, bcl-2,XIAP	geldanamyci, vorinostat	CD138+			3.2.6
Apigenin 7-O-(4″-O-p-E-coumaroyl)-glucoside	apoptosis	AnnV					3.2.6
Arctiin	JAK/STAT3	bcl-2, VEGF, MMP-2, PTPepsilon	BTZ				3.2.2
Asiaticoside	STAT3, autophagy						3.3.3
Baicalein	E3-ubiquitin ligase complex	CRBN, IKFZ 1 and 3					3.2.6
Berberine	miRNA screens, cell cycle	miRNA21, PDCD4					3.1.1
Berberine	miRNA screens	miR-99a-125b, miR-17–92, miR-106–25					3.1.1
Berberine	miRNA screens	miR-106b-25, p38 MAPK					3.1.1
Bergamottin	STAT3	STAT3					3.2.3
Betulinic acid	NF-kB, MMP	NF-kB, ROS, bcl-2, bax			Nude mice		3.3.3
Betulinic acid	STAT3						3.3.3
Bigelovin	block E2F1	cyclin D, E, A, CDK4		CD138+			3.3.1
Bruceantin	MMP	c-myc			Scid mice	MMP	3.3.3
Bruceantin	Migration, Notch	Notch				MM-CSC	3.3.3
Butein	STAT3	bcl-2, SHP, c-Src					3.2.5
Capillarisin	STAT3	STAT3 location					3.2.3
Capsaicin	STAT3, cell cycle	STAT3, JAK, Src, bcl-2, bcl-XL, survivin, VEGF	BTZ, Thal		Athymic nude mice		3.1.2
Cardamonin	NF-kB	bcl-2, bcl-XL					3.2.5
Celastrol	NF-kB, MMP						3.3.3
Cnicin	apoptosis	NF-kB, ROS, Pim-2	AKT-inhibitor, Mel, BTZ	BMMC: MM CD38++/45-	Chicken CAM assay	stroma cells	3.3.1
Coleon G	Proliferation					MM-CSC	3.3.2
Coleon O	proliferation					MM-CSC	3.3.2
Crocin	JAK2/STAT3	SHP-1, blc-2, CXCR4, VEGF					3.3.4
Curcumin	STAT1/3	STAT1/3	Dex				3.2.3
Curcumin	apoptosis	Caspases 3/9, bcl-2					3.2.3
4′-O-demethyldioncophylline A	apoptosis	AnnV					3.1.1
5′-O-demethyldioncophylline A	apoptosis	AnnV					3.1.1
4′-O-demethyl-7-epi-dioncophylline A	apoptosis	AnnV					3.1.1
Diallyl trisulfide	apoptosis, clonogenic assay	AnnV				MM-CSC	3.1.2
Dihydrocaffeic acid	proteasome activity	ROS, MMP	* inhibits BTZ	CD138+			3.2.2
2,4-dihydroxy-3′-methoxy-4′-ethoxychalcone	PI3K/p-AKT/mTOR	bcl-2, bad					3.2.5
5,3′-dihydroxy-3,6,7,8,4′-pentamethoxyflavone	proliferation		BTZ, Thal				3.2.6
Dioncophylline A	apoptosis	AnnV					3.1.1
Dioncophylline C	apoptosis	AnnV					3.1.1
Dioncophylline D_2_	apoptosis	AnnV					3.1.1
Dioncophylline F	apoptosis	AnnV					3.1.1
Dioncoquinone A	apoptosis	AnnV					3.2.1
Dioncoquinone B	apoptosis	AnnV					3.2.1
Dioncoquinone C	apoptosis	AnnV					3.2.1
Dioncoquinone D	apoptosis	AnnV					3.2.1
Dioncoquinone E	apoptosis	AnnV					3.2.1
Emodin	JAK2/STAT3	Mcl-1, gp130					3.2.1
Epigallocatechin gallate	MMP	ROS	* inhibits BTZ	CD138+			3.2.8
Fangchinolin	apoptosis, NF-kB	bcl-2, bcl-XL, p65, AP-1,survivin, COX2					3.1.1
Formononetin	STAT3/5,	bcl-2, VEGF, ROS	BTZ		Nude mice		3.2.7
Forskolin	viability	bim	Dox, Dex, Mel Btz, Cyclophos	CD138+			3.3.2
Gallic acid	MMP	ROS	* inhibits BTZ	CD138+			3.2.8
Gambogic acid	CXCR4, migration, invasion	NFkB				Osteoclas s (murine)	3.2.7
Gambogic acid	Hypoxia, PI3K/AKT/mTOR	HIF-1a			Balb/c nude mice		3.2.7
Gambogic acid	ROS	Sirt1					3.2.7
Genistein	NF-kB	miRNA-29b					3.2.7
Genistein	apoptosis	caspase 3					3.2.7
Genistein	NF-kB	ICAM1, bcl-2, bcl-XL					3.2.7
Glucomoringin	NF-kB	NF-kB			Nude mice		3.1.2
Glucoraphanin	NF-kB	NF-kB			Nude mice		3.1.2
Gossypol	apoptosis	bcl-2, bcl-XL					3.3.1
Gossypol	IL-6 induced JAK2/STAT3	Mcl-2, bcl-2					3.3.1
Honokiol	osteoclastogenesis, NF-kB	NF-kB				osteoclasts	3.2.2
8-hydroxycalamene	STAT3	caspase3/9, bcl-2, PIAS3	BTZ				3.3.1
Icaritin	JAK2/STAT3, JNK/ERK	IL-6		CD138+	NOD/SCID mice	BM-stroma cells	3.2.6
Isobavachalcone	autophagy	LC3-II	chloroquine, beclin shRNA				3.2.5
Jozimine A_2_	viability						3.1.1
Komaroviquinone	cytotoxicity						3.3.2
Luteolin	autophagy	LC3-II					3.2.6
Lycorine	Wnt/b-catenin	ALDH1, c-myc, CCDN, GLI, SMO, Notch	Pom, Dox, Mel, BTZ	CD138+		MM-CSC	3.1.1
Mangiferin	viability						3.2.7
Matrine	AKT	p-AKT, Casp3, PARP, bim, bcl-2, survivin	Arsenic trioxide				3.1.1
25-O-methylcimigenol-3-O-α-L-arabinopyranoside	apoptosis	AnnV					3.3.3
25-O-methylcimigenol-3-O-β-D-xylopyranoside	apoptosis	AnnV					3.3.3
5′-O-methyldioncophylline D	apoptosis	AnnV					3.1.1
Miyabenol C		MMP					3.2.4
Naringenin	apoptosis	AnnV					3.2.6
Oleacein	epigenetic, cell cycle	acetylated histons, histon deacetylase, Sp-1, p27, p21	Carfilzomib	CD138+		stroma cells	3.2.2
Oridonin	pAKT	PTEN				CSC of RPMI8226/ BTZres	3.3.2
Parthenolide	NF-kB	c-FLIP	Dex, TRAIL	CD138+			3.3.1
Parthenolide	proliferation					MM-CSC, stroma, and ECM	3.3.1
Pentagalloyl glucose	myc inhibition		* inhibits BTZ				3.2.8
Plumbagin	apoptosis	AnnV					3.2.1
Pristimerin	Proteasome	NF-kB		CD138+			3.3.3
Psorospermin	mdr1/P-glycoproptein						3.2.7
Pterostilbene	proliferation	AnnV, MMP, caspase 3/9, p-ERK, JNK		CD138+	NOD/SCID mice		3.2.4
Pterostilbene	proliferation	p-AKT, p-p38, MMP					3.2.4
Quercetin	MMP	ROS	* inhibits BTZ	CD138+			3.2.6
Resveratrol	NF-kB, osteoclast diff. and resorption	RANKL					3.2.4
Resveratrol	MMP						3.2.4
Resveratrol	ROS	SMAC					3.2.4
Resveratrol	UPR	lncRNA NEAT1					3.2.4
Resveratrol	Invasion	MMP-2, MMP-9, bcl-2, bcl-XL					3.2.4
Resveratrol	STAT3, NF-kB, p-AKT	bcl-2, bcl-XL, bax	BTZ, Thal	CD138+			3.2.4
β-sesquiphellandrene	apoptosis	bcl-2					3.3.1
Tannic acid	MMP	ROS	* inhibits BTZ	CD138+			3.2.8
Tanshinone II_A_	Autophagy	LC3-II					3.3.2
Theaflavin digallate	ER stress, proliferation	ER-stress reporter protein; splicing of XBP1					3.2.8
Thymoquinone	STAT3, p-AKT	bcl-2, bcl-XL	Thal, BTZ				3.3.4
Ursolic acid	Inhibition of deubiquitination	USP7 activity, MDM2, DNMT1					3.3.3
ε-viniferin	MMP	MMP					3.2.4
Withaferin A	Proliferation	HEK cells/NF-kB -reporter				MM-CSC	3.3.3
Withanolide F	Proliferation	HEK cells/NF-kB -reporter				MM-CSC	3.3.3
Wogonin	p-AKT	Bax					3.2.6
